# 3KO-NSCs ameliorate behavioral deficits and modulate gut microbiota in a VPA-induced C57BL/6 mouse model of autism

**DOI:** 10.3389/fimmu.2025.1680179

**Published:** 2025-10-02

**Authors:** Caixia Wu, Xianjie Li, Han Wang, Xiaoya Yang, Zhaoming Liu

**Affiliations:** ^1^ Institute of Biological and Medical Engineering, Guangdong Academy of Sciences, Guangzhou, China; ^2^ National Engineering Research Center for Healthcare Devices, Institute of Biological and Medical Engineering, Guangdong Academy of Sciences, Guangzhou, China; ^3^ Department of Physiology, Guangzhou Health Science College, Guangzhou, China; ^4^ Guangzhou Institutes of Biomedicine and Health, Chinese Academy of Sciences, Guangzhou, China; ^5^ University of Chinese Academy of Sciences, Beijing, China

**Keywords:** 3KO-NSCs, autism spectrum disorder (ASD), VPA, neuroinflammation, microbiota dysbiosis

## Abstract

**Background:**

Autism spectrum disorder (ASD) involves complex neurological and gastrointestinal pathophysiology. Existing therapies rarely address the gut-brain axis connection. This study evaluated the therapeutic potential of immune-evasive human induced pluripotent stem cell-derived neural stem cells (3KO-NSCs) in a mouse model of ASD.

**Methods:**

We used a valproic acid (VPA)-induced ASD model in C57BL/6 mice. Mice received systemic administration of 3KO-NSCs. Assessments included behavioral assays (social interaction, repetitive behaviors), hippocampal cytokine profiling (IL-6, TNF-α), 16S rRNA sequencing for gut microbiota analysis, immunohistochemistry (Iba1^+^ microglia), and ultrastructural synaptic analysis.

**Results:**

3KO-hiPSC-NSC treatment significantly ameliorated VPA-induced ASD-like behaviors. It reduced hippocampal neuroinflammation (decreased IL-6 and TNF-α) and attenuated microglial overactivation (reduced Iba1+ cells), correcting synaptic pruning abnormalities. Concurrently, treatment restored gut microbiota diversity (increased Shannon index), enriching Bacteroides and reducing pro-inflammatory Proteobacteria.

**Conclusions:**

3KO-NSCs exert dual therapeutic effects by mitigating central neuroinflammation and rebalancing gut microbiota. This provides the first direct evidence that stem cell therapy can modulate the gut-brain axis to treat ASD, positioning 3KO-NSCs as a novel bifunctional therapeutic strategy.

## Highlights

Key findings demonstrate that CRISPR-edited 3KO-NSCs simultaneously ameliorate ASD-like behaviors by:

- reducing hippocampal neuroinflammation (IL-6/TNF-α↓) and microglial activation (Iba1+↓).
- restoring gut microbiota diversity (↑Bacteroides/↓Proteobacteria).

## 1 Introduction

Autism spectrum disorder (ASD) represents a profound global health challenge, affecting approximately 1 in 54 children and imposing lifelong socioeconomic burdens exceeding $3.6 million per individual (1, 2). This complex neurodevelopmental condition, characterized by social communication deficits and repetitive behaviors, arises from intertwined neurological and gastrointestinal pathophysiology (3–5). Mounting evidence implicates dysregulation of the gut-brain axis—a bidirectional communication network linking enteric microbiota with central nervous system (CNS) function—as a critical etiological factor (6). Seminal studies demonstrate that fecal microbiota transplants from ASD donors induce behavioral deficits in germ-free mice, establishing causal proof of microbial contributions to ASD pathogenesis (7–9). Concurrent multi-omics analyses reveal ASD-specific microbial signatures, including reduced Firmicutes/Bacteroidetes ratios and diminished short-chain fatty acid (SCFA) production, particularly butyrate. These perturbations coincide with neuroinflammatory cascades, synaptic dysfunction, and behavioral abnormalities in preclinical models, underscoring the multifactorial nature of ASD.

Despite decades of research, current pharmacological interventions (e.g., risperidone, aripiprazole) merely alleviate associated symptoms without addressing core pathologies (10, 11). The valproic acid (VPA)-exposed C57BL/6 mouse model has emerged as a gold standard for ASD preclinical studies, reliably recapitulating social deficits (significantly reduction in three-chamber tests), repetitive behaviors (significantly increased self-grooming), hippocampal neuroinflammation, and prefrontal cortex hypoconnectivity (12, 13). Parallel advances in stem cell therapy highlight human induced pluripotent stem cell-derived neural stem cells (hiPSC-NSCs) as promising candidates for neurological disorders (14). Phase I/II trials confirm their safety in spinal cord injury and Parkinson’s disease, while preclinical studies demonstrate their capacity to secrete neurotrophic factors, suppress neuroinflammation, and enhance synaptic plasticity in ASD models (15, 16). However, two fundamental limitations persist: (1) Conventional NSC transplants face rapid immune rejection, with >80% graft loss within 8 weeks due to CD8+ T cell infiltration, necessitating toxic immunosuppression (17); and (2) Existing mono-mechanistic approaches fail to concurrently address neurological and gastrointestinal dimensions of ASD, neglecting gut-brain axis crosstalk.

Critical scientific and technical challenges remain unresolved. First, while gut microbiota dysbiosis correlates with ASD, causal mechanisms linking specific microbial metabolites (e.g., butyrate) to neuroinflammation or synaptic pruning are poorly defined. Second, immune rejection severely limits the translational potential of allogeneic hiPSC-NSCs, despite their regenerative capabilities. Although CRISPR/Cas9-engineered hypoimmunogenic stem cells—such as triple-knockout (3KO) designs targeting B2M, CIITA, and CD40—extend graft persistence, their efficacy in modulating the gut-brain axis remains unexplored. Third, current therapeutic paradigms focus narrowly on neural or microbial targets, overlooking synergistic interactions between CNS repair and microbiota normalization. This gap is exacerbated by technical constraints in monitoring multi-system responses, as few studies integrate behavioral, neuroinflammatory, ultrastructural, and microbiological endpoints within a unified framework.

To address these challenges, we pioneer a transformative strategy using immune-evading 3KO-NSCs to simultaneously target neuroinflammation and gut dysbiosis in a VPA-induced C57BL/6 mouse ASD model. Building on Stubbendorff et al.’s hypoimmunogenic platform, we engineer B2M/CIITA/CD40-null hiPSC-NSCs (18). We hypothesize that these cells exert dual therapeutic effects by: (i) Attenuating hippocampal neuroinflammation via NF-κB pathway inhibition (reducing Iba1^+^ microglia); and (ii) Restoring gut microbiota homeostasis through tryptophan metabolism modulation (increasing Bacteroides abundance). Our study reveals a novel mechanistic triad: 3KO-NSCs enhance synaptic plasticity, upregulate microbiota-derived butyrate, and reduce systemic inflammation (IL-6 ↓). This bifunctional approach transcends historical cell therapy barriers, representing the first experimental evidence that engineered stem cells can systemically remodel ASD pathophysiology via gut-brain axis modulation.

By unifying hypoimmunogenic stem cell engineering with multi-omics validation of gut-brain interactions, this work establishes a paradigm-shifting therapeutic framework for neurodevelopmental disorders. Beyond ASD, it offers a blueprint for combinatorial therapies targeting interconnected neurological and gastrointestinal pathologies.

## 2 Materials and methods

### 2.1 Experimental design and animal procedures

All procedures were approved by the Institutional Animal Care and Use Committee (IACUC) of the Guangzhou Institute of Biomedicine and Health, Chinese Academy of Sciences (Approval No. IACUC:2023081) and complied with AAALAC-accredited SPF standards and institutional guidelines. This study follows the ARRIVE guidelines (Percie du Sert et al., 2020) and strictly adhered to the Guide for the Care and Use of Laboratory Animals (National Academies Press, 8th edition, 2011). Euthanasia and anesthesia conformed to the 2020 AVMA Guidelines for the Euthanasia of Animals, excluding prohibited agents (e.g., chloral hydrate, ether, chloroform). Animal suffering was minimized using appropriate analgesics and humane endpoints.

#### 2.1.1 Animal housing and breeding

Adult C57BL/6 mice (12 males, 24 females; initial body weight 20–25 g) were housed under controlled conditions (22 ± 1°C, 55 ± 5% relative humidity) with a 12-hour light/dark cycle (lights on 07:00). Animals received autoclaved water and standard rodent chow ad libitum. For mating, male and female mice were co-housed at 17:00; vaginal plugs were checked at 09:00 the following morning. Plug-positive females were designated as gestational day (GD) 0.5.

#### 2.1.2 Prenatal VPA exposure

Twenty-four confirmed pregnant dams were randomly assigned via stratified randomization to two groups. During peak neocortical neurogenesis (GD11.5 to GD13.5), dams in the VPA-exposed C57BL/6 mice group (n = 16) received daily intraperitoneal injections of sodium valproate dissolved in endotoxin-free saline (300 mg/kg on GD11.5, 400 mg/kg on GD12.5, and 300 mg/kg on GD13.5). Control dams (n = 8) received equivalent volumes of 0.9% NaCl.

#### 2.1.3 Postnatal group allocation

Male offspring were cross-fostered to minimize litter effects. At postnatal day 21 (P21), offspring were randomly assigned to three experimental groups: Control (n = 12; saline-treated offspring from control dams), VPA (n = 12; VPA-exposed C57BL/6 mice offspring), and 3KO-hiPSCs (n = 12; VPA-exposed C57BL/6 mice offspring receiving 3KO-NSCs). Experimenters were blinded to group assignments during both behavioral testing and subsequent biochemical analyses. Behavioral assessments were followed by histological evaluations.

#### 2.1.4 Experimental timeline and treatment schedule

Prenatal VPA exposure was conducted via intraperitoneal injections administered on gestational days (GD) 11.5, 12.5, and 13.5 at doses of 300, 400, and 300 mg/kg, respectively. Male offspring were randomly assigned to experimental groups at postnatal day (PND) 21. A combinatorial treatment regimen was initiated at PND21 and continued weekly for four weeks, consisting of systemic administration of 3KO-NSCs (2×10^6^ cells per injection) via tail vein injection accompanied by intranasal exosome (Exos) instillation on PND21, 28, 35, and 42. Additionally, a bilateral intracerebroventricular boost of 3KO-NSCs was performed using stereotactic surgery on PND42. Behavioral assessments began on PND48 with fecal sample collection for 16S rRNA sequencing, followed by the open field test on PND49–50. The self-grooming and marble-burying tests were conducted on PND51–52, and the three-chamber social test—including both habituation and testing phases—took place from PND53 to 59. The Morris water maze assay was then carried out from PND60 to 67. Finally, animals were euthanized between PND68 and P70 for tissue collection and subsequent biochemical and morphological analyses.

### 2.2 Characterization of 3KO-hiPSCs pluripotency and neuronal differentiation potential

#### 2.2.1 RT-qPCR

Total RNA was extracted from cells using Trizol reagent, purified by chloroform extraction and isopropanol precipitation, and reverse-transcribed into cDNA. Quantitative PCR amplification was performed using gene-specific primers (sequences below), SYBR Green-based PCR master mix, and a real-time PCR system with fluorescence signal detection. Relative gene expression levels were calculated using the 2-ΔΔCT method, normalizing to GAPDH as the internal reference. The following primers were used:hGapdh F: АСАСССАСТССТССАССТТТ, R: TTACTCCTTGGAGGCCATGT;hNanog F: TGAACCTCAGCTACAAACAG, R: TGGTGGTAGGAAGAGTAAAG;hOct4 F: CCTCACTTCACTGCACTGTA, R: CAGGTTTTCTTTCCCTAGCT;hSox2 F: CCCAGCAGACTTCACATGT, R: CCTCCCATTTCCCTCGTTTT;hPax6 F: TTGCTTGGGAAATCCGAG, R: TGCCCGTTCAACATCCTT;hNestin F: CCACCCTGCAAAGGGAATCT, R: GGTGAGCTTGGGCACAAAAG.

#### 2.2.2 Flow cytometry analysis

Harvested cells were washed twice with cold PBS and resuspended in staining buffer (PBS containing 1% FBS). Cell suspensions were incubated with fluorochrome-conjugated antibodies targeting specific surface markers (e.g., CD34, CD45) for 30 minutes at 4°C in the dark. After washing with staining buffer, cells were fixed with 4% paraformaldehyde for 15 minutes at room temperature. Samples were acquired on a BD FACS Calibur flow cytometer using appropriate compensation controls (single-stained samples), and data were analyzed using FlowJo software.

#### 2.2.3 Karyotype analysis

Karyotype analysis followed a standard protocol. Briefly, cells at 80-90% confluency were incubated with fresh medium containing 20 μg/ml colcemid for 2 hours to arrest them at metaphase. Arrested cells were trypsinized, collected by centrifugation, resuspended in hypotonic solution, and incubated at 37°C for 30 minutes. Cells were then fixed in methanol acid (3:1), dropped onto pre-chilled glass slides, and air-dried overnight. Slides were stained with 1% Giemsa solution for 10 minutes. Karyotypes were analyzed microscopically, and images were captured for evaluation.

#### 2.2.4 Teratoma formation assay

Pluripotency of 3KO-hiPSCs was assessed by teratoma formation. 1×10^6^ cells suspended in 100 μL PBS mixed with an equal volume of Matrigel were injected subcutaneously into the dorsal flanks of 6–8 week-old immunodeficient SCID mice. After 6–8 weeks, resulting tumors were excised, fixed in 4% paraformaldehyde, and embedded in paraffin. Histological analysis via hematoxylin and eosin (H&E) staining identified tissues derived from ectoderm, mesoderm, and endoderm. All procedures were approved by the IACUC and conducted according to institutional ethical guidelines.

#### 2.2.5 Cell culture and differentiation of iPSC-derived neurons

3KO-hiPSCs were cultured on Matrigel (5 μL/mL; Corning) in mTeSR™1 medium (Stemcell Technologies). After 3 days, cells were re-plated onto Matrigel-coated 12-well plates in N_2_B_27_ medium supplemented with 2i inhibitors. The N_2_B_27_ + 2i medium comprised a 1:1 mixture of N_2_ medium (1×N_2_, DMEM/F-12, 1× NEAA, 1× GlutaMAX, 5 μg/mL insulin, 1 mM L-glutamine, 100 μM 2-mercaptoethanol) and B_27_ medium (Neurobasal Medium, 1×B_27_, 5 μM SB431542, 5 μM dorsomorphin). After 7 days, cells were re-plated onto Matrigel-coated 6-well plates in N_2_B_27_ medium. Visible neural rosettes were picked, dissociated into single cells using accutase (Sigma), and re-plated onto Matrigel-coated 24-well plates in N_2_B_27_ medium for neuronal differentiation (>1 month).

#### 2.2.6 Electrophysiological recordings

Whole-cell voltage- or current-clamp recordings were performed using 6–9 MΩ borosilicate glass electrodes, with specific protocols detailed in corresponding figures. Signals were amplified using an Axopatch 200B amplifier (Axon Instruments) and acquired/analyzed with Clampfit 10.2 software (Molecular Devices). Pipettes (4–8 MΩ resistance when filled) contained (in mM): 140 potassium methanesulfonate, 10 HEPES, 5 NaCl, 1 CaCl_2_, 0.2 EGTA, 3 ATP-Na_2_, 0.4 GTP-Na_2_ (pH 7.2, KOH-adjusted). The bath solution contained (in mM): 127 NaCl, 3 KCl, 1 MgSO_4_, 26 NaHCO_3_, 1.25 NaH_2_PO_4_, 10 D-glucose, 2 CaCl_2_ (pH 7.4, NaOH-adjusted). Cells on coverslips were maintained in oxygenated (95% O_2_/5% CO_2_) bath solution at room temperature during recordings.

#### 2.2.7 Tumor formation assay of 3KO-NSCs

To assess tumorigenicity, 1×10^7^ 3KO-NSCs were injected into either the left quadriceps muscle or subcutaneous abdominal space of 8-week-old NOD-SCID mice. After 6 months, mice were euthanized for histological examination of muscles, skin, liver, kidneys, myocardium, small intestine, brain, spleen, and lungs. All procedures followed institutional ethical guidelines.

### 2.3 3KO-NSCs transplantation protocol

#### 2.3.1 Exosome isolation and characterization

Exosomes were isolated from human umbilical cord mesenchymal stem cell (hUC-MSC) conditioned media by differential ultracentrifugation following MISEV2018 guidelines. Supernatants were sequentially centrifuged (Eppendorf 5910 R) at: 300 ×g for 10 min (4°C) to remove cells, 2,000 ×g for 10 min (4°C) to clear debris, and 10,000 ×g for 30 min (4°C) to pellet microvesicles. Clarified supernatant was filtered (0.22-μm PES membrane; Millipore) and ultracentrifuged at 100,000 ×g for 2 h (4°C; Optima XPN-100, Beckman Coulter). Purified exosome pellets were resuspended in sterile PBS for downstream use.

#### 2.3.2 Combinatorial therapy administration

The 3KO-hiPSC group received therapy via two routes:

1. Systemic Delivery: Weekly IV injections of 2×10^6^ 3KO-NSCs (P3-5, >95% viability) in 200 μL PBS via tail vein for 4 weeks.
2. Intracerebroventricular (ICV) Boost: At week 4 post-systemic treatment, stereotactic surgery under 3% isoflurane anesthesia injected 2 μL (1×10^5^ cells/μL) bilaterally into lateral ventricles (0.4 μL/min flow rate; coordinates from bregma: AP -1.0 mm, ML ±0.8 mm, DV -1.5 mm). Needles were withdrawn after 10 minutes. Intranasal administration of 50 μg exosomes in 20 μL PBS followed, delivered via micropipette tip inserted 2 mm into the nasal cavity. Animals received 0.3% gentamicin peri-transplantation to mitigate infection risk. Behavioral testing commenced 1 week post-treatment.

### 2.4 Behavioral tests

C57BL/6 mice were acclimated to the experimental room for 1 hour before testing.

#### 2.4.1 Three-chamber social interaction test

The three-chamber social interaction test was performed to assess sociability and social novelty preference. Prior to testing, all mice underwent a habituation phase consisting of daily 2-hour acclimation sessions under controlled conditions (23°C, 50 lux) on days 1–7, followed by central compartment exploration and full arena access for 3 minutes each on days 8–10. The experimental sequence began with a 5-minute baseline session where both lateral chambers contained empty wire cages. This was followed by a 10-minute sociability test, in which mice were presented with a choice between an age- and sex-matched stranger mouse (Stranger 1) and an empty cage. Finally, a 10-minute social novelty test was conducted by introducing a novel unfamiliar mouse (Stranger 2) alongside the now-familiar Stranger 1. To minimize external variability, all stranger mice were individually housed for 48 hours before testing. Social interactions were automatically tracked using EthoVision XT 15.0 software, with an interaction defined as the test mouse’s snout being within 2 cm of the cage.

#### 2.4.2 Self-grooming test

Spontaneous self-grooming behavior was assessed to quantify repetitive and stereotypic movements. Each mouse was placed individually into a clean cage under standardized lighting conditions (50 lux). Following a 10-minute habituation period to minimize novelty-induced stress, spontaneous grooming behavior was recorded for 10 minutes. Video recordings were subsequently analyzed by three independent, blinded observers to quantify two key parameters: bout frequency, defined as discrete grooming episodes, and cumulative duration, reflecting the total time spent in stereotypic movements.

#### 2.4.3 Marble-burying test

Mice were acclimated for 3 min in chambers with 5 cm corncob bedding. Sixteen marbles (16 mm diameter) were arranged in a 4×4 grid (3 cm spacing). After 10 min testing (50 lux), three blinded observers scored marbles as buried if ≥75% covered by bedding (≥2/3 observer agreement; ambiguous cases resolved frame-by-frame).

#### 2.4.4 Open field test

Animals explored a 50 × 50 × 30 cm arena with a central zone (30 × 30 cm) for 10 min after 10 min habituation. An overhead camera and EthoVision XT software recorded:Central/peripheral zone crossings (all limbs crossing borders);Vertical activity (forelimbs raised ≥2 s).

#### 2.4.5 Morris water maze

Apparatus: Black pool (120 cm Ø) with opaqued water (23.0 ± 0.5°C); hidden platform (8 cm Ø; 1.5 cm below surface) in target quadrant.

##### 2.4.5.1 Acquisition (5 days)

4 trials/day (90 s max; 25 min ITI; randomized entry points); Platform-guided orientation for failed trials.

##### 2.4.5.2 Probe trial (24 hr post-training)

60 s free swim without platform;Spatial cues: Four high-contrast geometric patterns;Tracking: EthoVision XT v15.0 (<2% positional error).

### 2.5 Tissue collection and processing

Following behavioral testing, Cuthanasia was performed in accordance with the 2020 AVMA Guidelines for the Euthanasia of Animals. C57BL/6 mice were deeply anesthetized via intraperitoneal injection of ketamine (100 mg/kg) and xylazine (10 mg/kg). The absence of response to toe pinch and corneal reflex was used to confirm the surgical depth of anesthesia. This was followed by transcardial perfusion using 0.9% NaCl followed by 4% paraformaldehyde (PFA; Merck, Germany) for tissue fixation. Death was confirmed by the cessation of vital signs. Brains were then post-fixed in 4% PFA for 24 hr, after which the hippocampus and prefrontal cortex were dissected. Tissues were processed for paraffin embedding (Merck, Germany), and 5-μm coronal sections were cut using a rotary microtome (Microm HM335E, Thermo Scientific).

### 2.6 Biochemical assays

Oxidative stress markers (GSH, T-SOD, GSH-PX, MDA, CAT, T-NOS, NO) were quantified using commercial kits (Nanjing Jiancheng Institute of Biotechnology). Cytokine levels (IL-6, IL-10, IL-1β, TNF-α) were measured with mouse-specific ELISA kits (Fankel). All assays followed manufacturers’ protocols.

### 2.7 16S rRNA sequencing and bioinformatics analysis

#### 2.7.1 Sample collection and preservation

Fresh fecal samples were collected aseptically from C57BL/6 mice:

1. Mice were briefly restrained; defecation was induced via gentle tail elevation and abdominal massage.
2. Fresh pellets were transferred with sterile forceps into pre-labeled 2-mL tubes (Axygen, PCR-02-C).
3. Each tube contained 3–5 intact pellets (≈100 mg total).
4. Collections occurred daily between 09:00-11:00 to minimize circadian effects.
5. Specimens were flash-frozen in liquid nitrogen within 5 min and stored at -80°C until analysis.

#### 2.7.2 DNA extraction and 16S rRNA amplification

##### 2.7.2.1 Genomic DNA isolation

DNA was extracted using a modified CTAB protocol with liquid nitrogen homogenization (0.1 mm zirconium beads; FastPrep-24). Purity was verified by NanoDrop 2000 (A_260_/A_280_ = 1.8–1.9; A_260_/A_230_ > 2.0). DNA was normalized to 1 ng/μL with ultrapure water.

##### 2.7.2.2 V3-V4 amplification

Target regions were amplified using barcoded primers 341F/806R with Phusion^®^ Master Mix. Cycling: 98°C/1 min; 30 cycles of 98°C/10 s, 50°C/30 s, 72°C/30 s; final extension 72°C/5 min.

#### 2.7.3 Library preparation and sequencing

##### 2.7.3.1 Amplicon purification

PCR products (≈550 bp) were size-verified via 2% agarose gel electrophoresis and purified (TIANgel Midi Kit).

##### 2.7.3.2 Library construction

Libraries were prepared using NEBNext Ultra II Kit. Quality control included size distribution (Agilent 5400 Bioanalyzer; 450–650 bp) and quantification (Qubit 4.0; ≥20 nM).

##### 2.7.3.3 Sequencing

Paired-end sequencing (PE 250) was performed on Illumina NovaSeq 6000 (50,000 reads/sample).

#### 2.7.4 Bioinformatics analysis

##### 2.7.4.1 Data processing

Raw sequences were denoised (QIIME2 v2023.2; DADA2) and taxonomically classified against SILVA 138.1.

##### 2.7.4.2 Diversity metrics

α-Diversity (Chao1, Shannon, Faith’s PD) and β-diversity (Bray-Curtis/UniFrac; PCoA/NMDS) were calculated.

##### 2.7.4.3 Differential taxa

LEfSe (LDA >2.0), ANCOM (W >0.7), and DESeq2 (FDR <0.05) were used.

##### 2.7.4.4 Functional profiling

KEGG pathways were predicted via PICRUSt2 v2.4.1; comparisons used Welch’s t-test with FDR correction in STAMP.

##### 2.7.4.5 Statistical modeling

Co-occurrence networks were constructed (Gephi v0.10.1; Spearman |ρ| >0.6, *P < 0.01*). Multivariate analyses included RDA (vegan) and PLS-DA (mixOmics).

### 2.8 Immunofluorescence staining

Brain sections were processed sequentially:

1. Deparaffinization and antigen retrieval were performed using standard protocols.
2. Sections were washed three times (5 min each) with PBS (pH 7.4).
3. Non-specific binding was blocked with 4% BSA in PBST (0.3% Triton X-100) for 1 hr at RT.
4. Primary antibodies (rabbit-derived, 1:200 in blocking solution) were incubated at 4°C for 16 hr under humidified conditions.
5. After incubation, sections were washed with PBST (3 × 10 min).
6. Secondary staining used Alexa Fluor 488-conjugated goat anti-rabbit IgG (1:500) for 1 hr at RT (dark conditions).
7. Nuclei were counterstained with DAPI (1 μg/mL, 10 min, dark).

### 2.9 Transmission electron microscopy

For ultrastructural analysis, brain tissue samples were prepared from PND70 C57BL/6 mice. Following terminal anesthesia via intraperitoneal injection of ketamine/xylazine (75:5 mg/kg), transcardiac perfusion was performed through the ascending aorta. This involved an initial vascular flush with 200 mL of ice-cold PBS (0.01 M Na/K-phosphate, pH 7.4), followed by primary fixation with 300 mL of a mixed aldehyde solution (2% paraformaldehyde and 2.5% glutaraldehyde in 0.1 M sodium cacodylate buffer, pH 7.4; Sigma–Aldrich #G5882). Subsequently, microdissection was conducted to isolate 1 mm³ samples from the prefrontal cortex and dorsal hippocampus (n = 3 per group; 6 regions per animal).

The post-processing protocol consisted of secondary fixation in the primary fixative for 20 hours at 4°C, followed by treatment with 1% OsO_4_ and 0.8% K_4_[Fe(CN)_6_] in 0.1 M cacodylate buffer for 2 hours. Samples then underwent dehydration through a graded ethanol series (50% to 100%), were transitioned in propylene oxide, and embedded in EPON™ 812 resin with polymerization carried out at 60°C for 48 hours.

### 2.10 Statistical analysis

Data were analyzed using GraphPad Prism and presented as mean ± SD. Between-group comparisons used one-way ANOVA with Tukey’s *post hoc* test. Statistical significance was set at *P < 0.05*.

## 3 Results

### 3.1 Characterization of 3KO-hiPSCs pluripotency and neuronal differentiation potential

**Figure 1:**
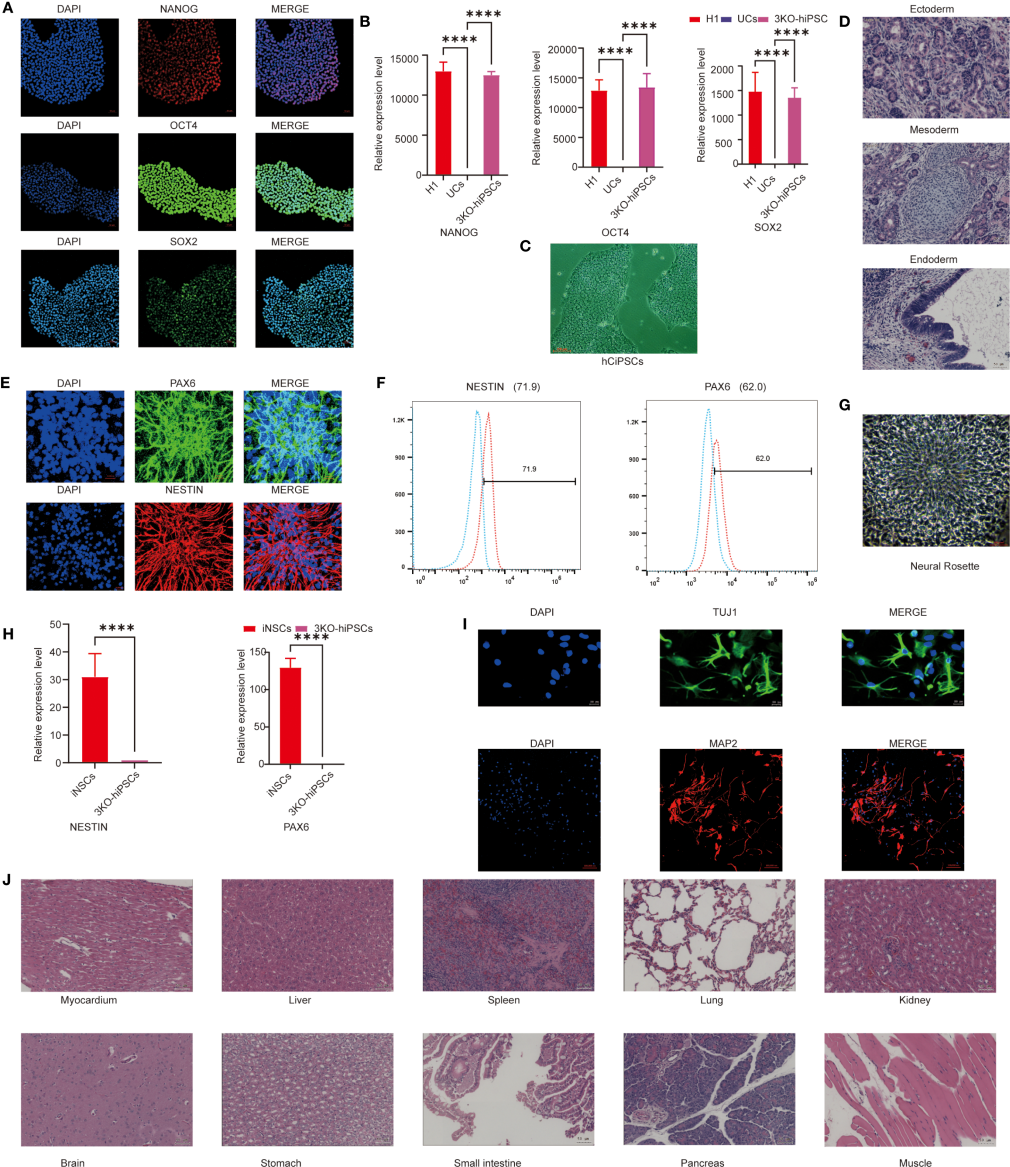
Characterization of 3KO-hiPSC pluripotency and neuronal differentiation potential. **(A)** Immunofluorescence staining of pluripotency markers (NANOG, OCT4, SOX2) in 3KO-hiPSCs. **(B)** RT-qPCR analysis of NANOG, OCT4, and SOX2 expression (*P < 0.0001* vs. control). **(C)** Phase-contrast microscopy of 3KO-hiPSCs. **(D)** H&E-stained teratoma sections showing derivatives of all three germ layers. **(E)** Immunofluorescence of NSC markers (PAX6, NESTIN) in 3KO-hiPSC-derived NSCs. **(F)** Flow cytometry quantification of PAX6^+^ (62%) and NESTIN^+^ (71.9%) populations. **(G)** Neural rosette structures in culture. **(H)** RT-qPCR of PAX6 and NESTIN in NSCs versus undifferentiated 3KO-hiPSCs (*P < 0.0001*; n = 3 biological replicates). **(I)** Immunofluorescence of neuronal markers (β-III-tubulin, MAP2) in differentiated neurons. **(J)** Histological analysis of major organs post-transplantation (scale bar: 50 μm).

To generate neural stem cells (NSCs) capable of long-term survival post-transplantation, we employed CRISPR/Cas9 to knockout B2M, CIITA, and CD40 genes in human urine-derived iPSCs, establishing low-immunogenicity triple-knockout hiPSCs (3KO-hiPSCs). Morphological assessment revealed that 3KO-hiPSCs resembled human embryonic stem cells (**Figure 1C**). Given this similarity, pluripotency was evaluated via immunofluorescence (IF) and RT-qPCR. IF confirmed high expression of NANOG, OCT4, and SOX2 (**Figure 1A**), while RT-qPCR demonstrated significantly elevated transcript levels of these genes (*P < 0.0001*; **Figure 1B**), collectively indicating embryonic stem cell-like pluripotency.

To assess differentiation potential, 3KO-hiPSCs were injected into murine axillary/hindlimb tissues. Teratomas formed within weeks, with H&E staining revealing structures from all three germ layers: endodermal epithelium, mesodermal chondrocytes, and ectodermal neural rosettes (**Figure 1D**), confirming embryonic stem cell-equivalent differentiation capacity.

We next characterized 3KO-hiPSC-derived NSCs (3KO-NSCs). IF showed high PAX and NESTIN expression (**Figure 1E**). Flow cytometry quantified PAX^+^ (62%) and NESTIN^+^ (71.9%) populations (**Figure 1F**). Neural rosettes were observed in culture (**Figure 1G**), and RT-qPCR confirmed significantly elevated PAX6/NESTIN expression versus undifferentiated 3KO-hiPSCs (*P < 0.0001*; **Figure 1H**). No tumors were detected in NOD-SCID mice 6 months post-transplantation of 1 ×10^7^ 3KO-NSCs across multiple organs (**Figure 1J**).

Further differentiation yielded neurons expressing early (MAP2) and mature (β-III-Tubulin) markers (**Figure 1I**). These results validate functional neuron derivation from 3KO-hiPSCs, providing a foundation for ASD mechanism studies using hiPSC-NSCs.

### 3.2 Open field test behavioral analysis

**Figure 2:**
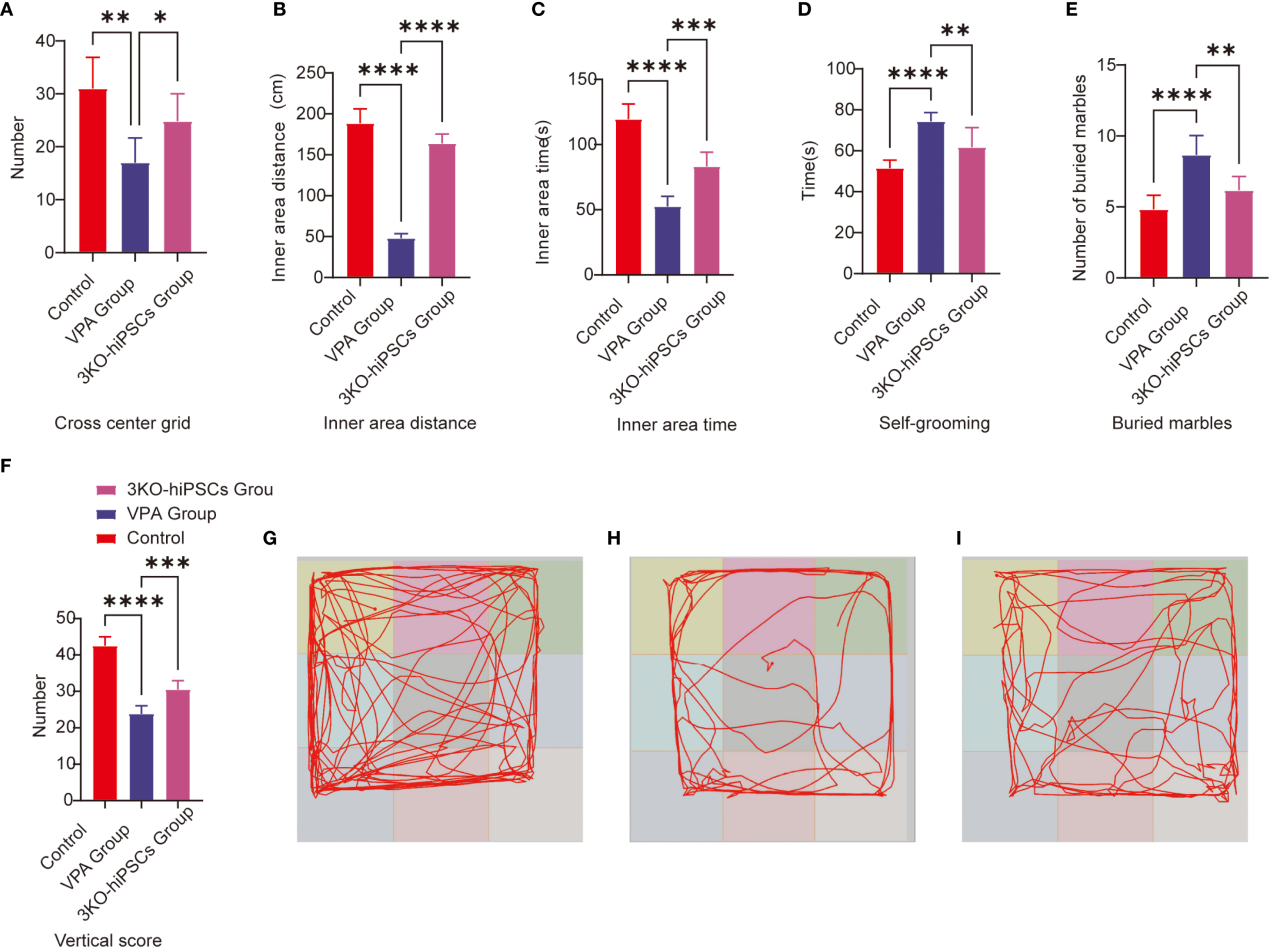
Behavioral analysis of C57BL/6 mice in the open field and repetitive stereotypic behaviors. **(A)** Cross-center grid; **(B)** inner area distance; **(C)** inner area time; **(D)** self-grooming; **(E)** buried marbles; **(F)** vertical score; **(G)** Representative open field trajectory map of the control group; **(H)** Representative open field trajectory diagram of the ASD model; **(I)** Representative open field trajectory maps of the 3KO-NSCs treatment group. The data are presented as the means ± SDs (x¯ ± s, n =12). Statistical significance: *P < 0.05, **P < 0.01, ***P < 0.001, ****P < 0.0001, compared with the VPA-exposed C57BL/6 mice ASD model group.

The open field test was employed to evaluate autonomous exploration and anxiety-like behaviors in a novel environment. Prenatal VPA exposure significantly suppressed exploratory behaviors and enhanced anxiety-like responses in C57BL/6 mice (**Figure 2**). Treatment with 3KO-NSCs ameliorated these deficits in the VPA-induced ASD model group.

Specifically, VPA-exposed mice exhibited significantly reduced center crossings compared to controls (P < 0.01; **Figure 2A**), which were increased following 3KO-NSCs treatment (P < 0.05). Similarly, the total movement distance in the inner area was markedly decreased by VPA (P < 0.0001 vs. control; **Figure 2B**), and this reduction was partially restored after intervention (P < 0.0001 vs. VPA group).

In terms of anxiety-like behaviors, VPA administration resulted in a significant decrease in time spent in the inner area (P < 0.0001; **Figure 2C**), which was prolonged by 3KO-NSCs treatment (P < 0.001). Vertical activity scores were also reduced in the VPA group (P < 0.0001; **Figure 2F**) and showed improvement after cell therapy (P < 0.001).

Trajectory analysis further supported these findings: VPA-exposed mice (**Figure 2H**) displayed restricted movement patterns with minimal center exploration, whereas the 3KO-NSCs-treated group (**Figure 2I**) exhibited trajectories resembling those of controls (**Figure 2G**), characterized by increased center exploration and reduced thigmotaxis.

### 3.3 Analysis of repetitive stereotypic behavior

#### 3.3.1 Buried marble test

The marble-burying test assessed repetitive behaviors in rodents. VPA exposure significantly increased marble-burying in C57BL/6 mice vs. controls (*P < 0.0001*; **Figure 2E**). 3KO-NSCs treatment reduced this behavior vs. the VPA group (*P < 0.01*), indicating partial normalization of exploratory patterns.

#### 3.3.2 Self-grooming

VPA-exposed C57BL/6 mice exhibited excessive self-grooming (**Figure 2D**), with higher frequencies vs. controls (*P < 0.0001*). 3KO-NSCs treatment significantly reduced grooming vs. the VPA group (*P < 0.01*).

### 3.4 Three-chamber social behavioral analysis

The three-chamber social test was employed to assess social motivation (sociability, 0–10 min) and social novelty preference (10–20 min) in experimental animals. This assay quantified social competence and cognitive flexibility by measuring exploration time directed towards unfamiliar conspecifics (Stranger 1 or Stranger 2) versus empty cages or objects.

#### 3.4.1 Social motivation phase (sociability, 0–10 min)

**Figure 3:**
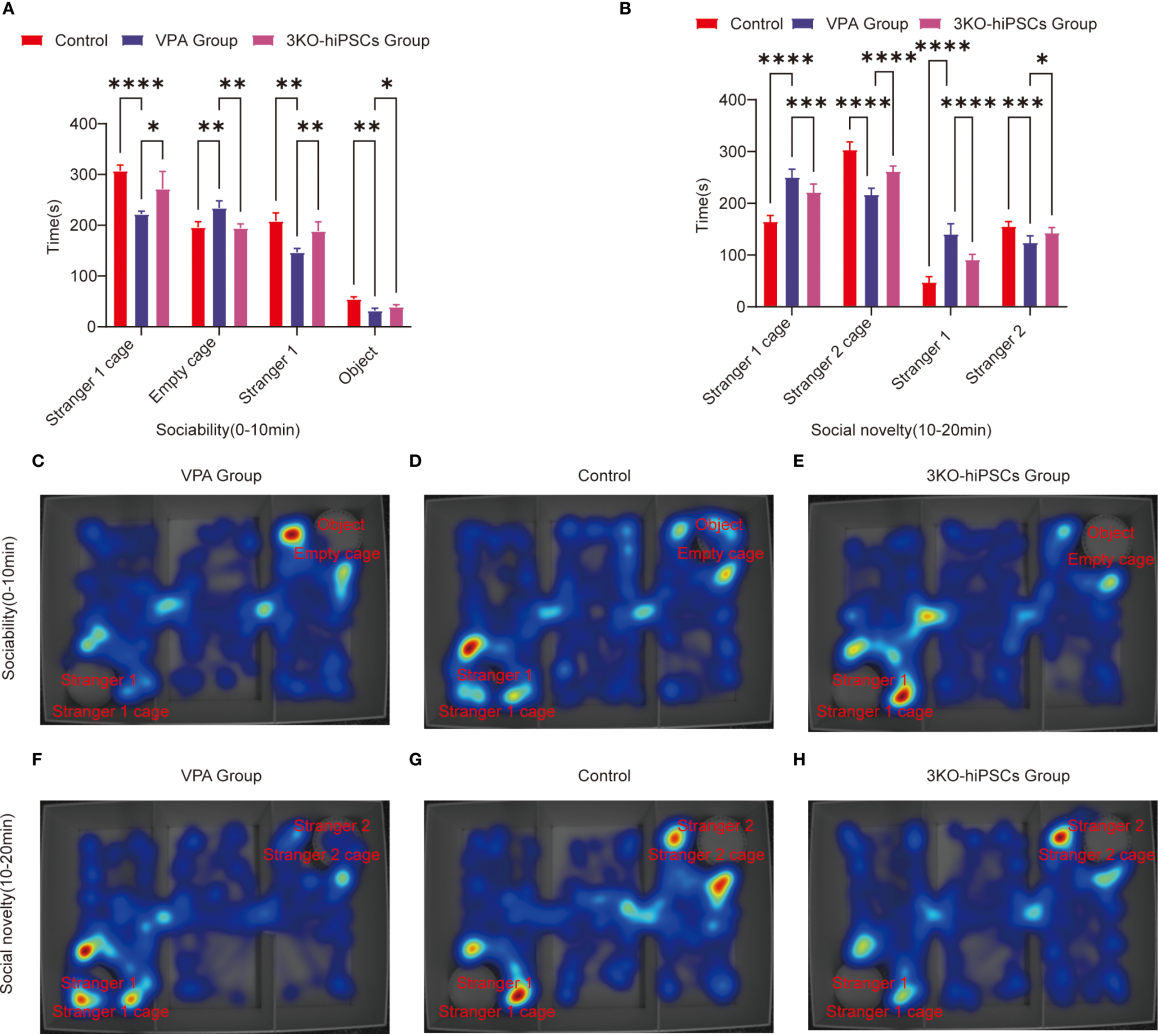
Behavioral analysis of three-chamber social interaction in C57BL/6 mice. **(A)** Sociability phase (0–10 min) showing interaction patterns with Stranger 1 versus empty cage. **(B)** Social novelty phase (10–20 min) demonstrating preference between familiar (Stranger 1) and novel (Stranger 2) conspecifics.Heat map representations: **(C)** ASD model group exhibited reduced exploration near Stranger 1 during sociability testing (0–10 min). **(D)** Control group displayed balanced exploration between social and nonsocial areas. **(E)** 3KO-NSCs treatment group showed restored social exploration patterns comparable to controls. **(F)** During social novelty testing (10–20 min), ASD model mice persistently occupied Stranger 1’s chamber. **(G)** Control mice distributed exploration time equally between Stranger 1 and Stranger 2. **(H)** 3KO-NSCs treated mice regained normal novelty-seeking behavior. Data expressed as mean ± SD (x¯ ± s, n =12). Statistical significance versus VPA-exposed ASD model group: *P < 0.05, **P < 0.01, ***P < 0.001, ****P < 0.0001. Metric definitions: Stranger 1/2 cage: Chamber occupancy time; Empty cage: Nonsocial area exploration Stranger 1/2: Direct sniffing/interaction duration; Object: Inanimate object investigation time.

VPA exposure significantly impaired social motivation in C57BL/6 mice (**Figure 3A**). Treatment with 3KO-NSCs ameliorated these deficits.

In the spatial exploration analysis, VPA-exposed C57BL/6 mice spent significantly less time near the Stranger 1 cage compared to controls (*P < 0.0001*, **Figure 3A**). 3KO-NSCs treatment significantly increased Stranger 1 exploration time relative to the VPA-exposed C57BL/6 mice group (*P < 0.05*, **Figure 3A**). Conversely, VPA-exposed C57BL/6 mice exhibited a pronounced preference for the empty cage versus controls (*P < 0.01*, **Figure 3A**), which was significantly reduced by 3KO-NSCs treatment (*P < 0.01*, **Figure 3A**).

Regarding social interaction behaviors, VPA-exposed C57BL/6 mice showed reduced sniffing time directed at Stranger 1 (*P < 0.01*, **Figure 3A**). 3KO-NSCs treatment significantly prolonged Stranger 1 sniffing time compared to the VPA group (*P < 0.01*, **Figure 3A**). While object sniffing time was also reduced in the VPA group (*P < 0.01*, **Figure 3A**), 3KO-NSCs treatment partially restored object exploration (*P < 0.05* vs. VPA group, **Figure 3A**).

Trajectory analysis (**Figures 3C–E**) revealed that the VPA-exposed C57BL/6 mice ASD model group (**Figure 3C**) actively avoided the Stranger 1 chamber while fixating on the empty cage. In contrast, the 3KO-NSCs treatment group (**Figure 3E**) displayed trajectories similar to controls (**Figure 3D**), characterized by increased proximity to Stranger 1 and reduced nonsocial exploration.

#### 3.4.2 Social novelty preference phase (10–20 min)

Analysis of spatial exploration patterns (**Figure 3B**) showed that VPA-exposed C57BL/6 mice spent excessive time near the Stranger 1 cage compared to controls (*P < 0.0001*). 3KO-NSCs treatment significantly reduced this hyperfixation (*P < 0.001* vs. VPA group). Simultaneously, VPA-exposed C57BL/6 mice avoided the novel Stranger 2 (*P < 0.0001*), an effect significantly counteracted by 3KO-NSCs treatment, which increased Stranger 2 exploration time (*P < 0.0001* vs. VPA group).

Social interaction disparities (**Figure 3B**) demonstrated abnormally high sniffing time directed at Stranger 1 in VPA-exposed C57BL/6 mice (*P < 0.0001*). 3KO-NSCs treatment attenuated this repetitive behavior (*P < 0.0001* vs. VPA group). Novel social exploration of Stranger 2 was suppressed in the VPA group (*P < 0.001*), and 3KO-NSCs treatment significantly enhanced Stranger 2 sniffing time (*P < 0.05* vs. VPA group).

Trajectory analysis (**Figures 3F–H**) showed the VPA-exposed C57BL/6 mice ASD model group (**Figure 3F**) persistently lingering near Stranger 1 while ignoring Stranger 2. The 3KO-NSCs treatment group (**Figure 3H**) exhibited dynamic exploration patterns resembling controls (**Figure 3G**), with balanced attention towards both Stranger 1 and Stranger 2.

### 3.5 Assessment of learning and memory capacity

The Morris water maze (MWM) assessed spatial learning and memory using acquisition trials (hidden platform training) and probe trials (memory retention testing).

#### 3.5.1 Spatial acquisition trials

**Figure 4:**
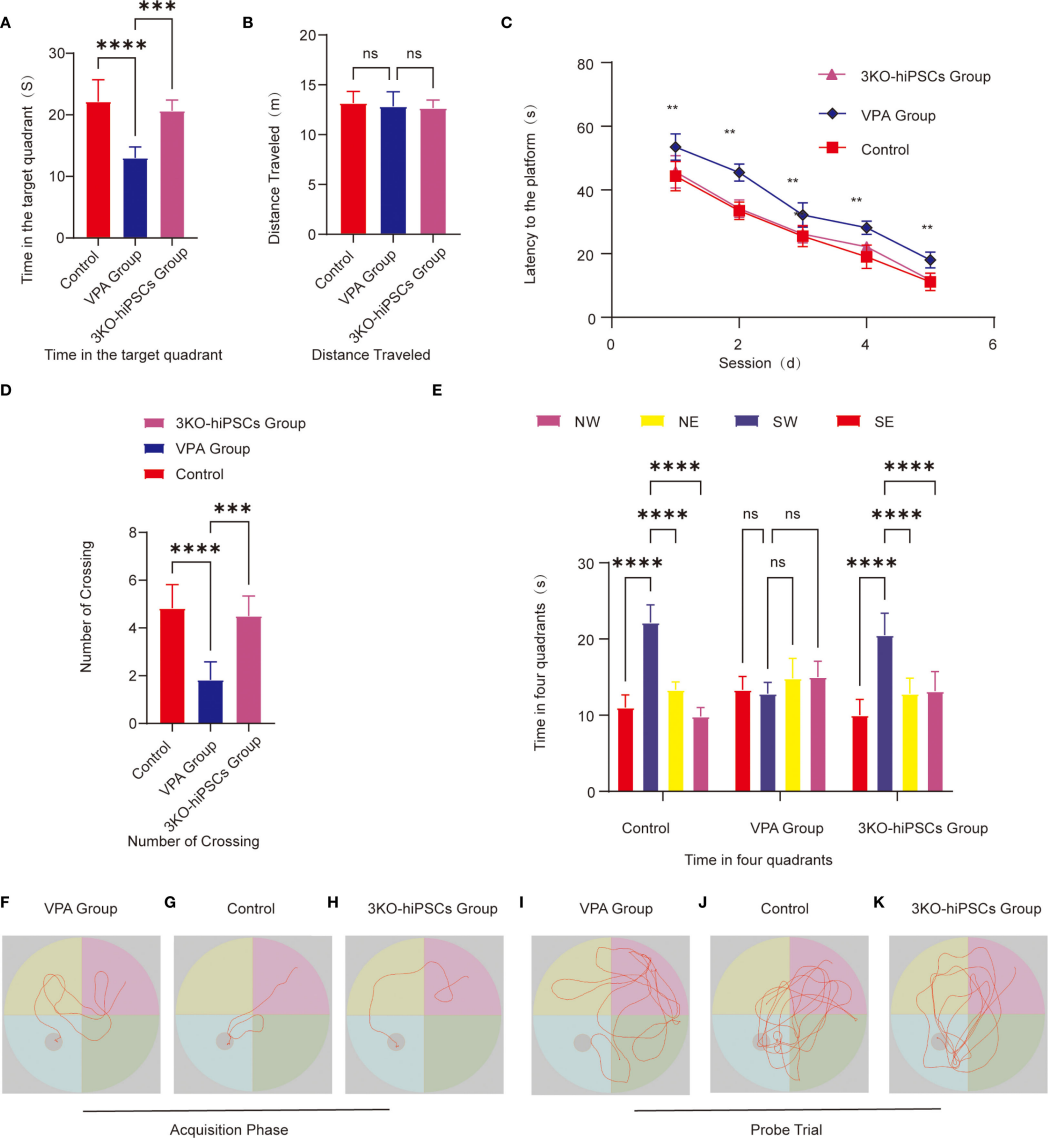
Morris water maze behavioral analysis. **(A)** Target quadrant duration. **(B)** Total swimming distance. **(C)** Escape latency to find the hidden platform. **(D)** Platform crossing frequency. **(E)** Quadrant time distribution during probe trials. Representative swimming paths during acquisition trials: **(F)** VPA-exposed C57BL/6 mice; **(G)** Control C57BL/6 mice; **(H)** 3KO-NSCs-treated C57BL/6 mice. Representative exploration trajectories during probe trials:**(I)** VPA-exposed group; **(J)** Control group; **(K)** 3KO-NSCs-treated group. Data presented as mean ± SD (x¯ ± s, n =12). Statistical significance versus VPA-exposed ASD model group: *P < 0.05, **P < 0.01, ***P < 0.001, ****P < 0.0001.

Escape latency (**Figure 4C**): VPA-exposed C57BL/6 mice showed significantly prolonged escape latencies compared to controls (*P < 0.01*), indicating impaired spatial learning. Treatment with 3KO-NSCs significantly reduced escape latencies (*P < 0.01* vs. VPA group), demonstrating accelerated task acquisition.

Search strategy (**Figures 4F–H**): The VPA-exposed group exhibited undirected, random swimming patterns (**Figure 4F**). In contrast, the control group showed goal-directed navigation with direct platform approaches (**Figure 4G**). The 3KO-NSCs-treated group displayed improved search efficiency, with trajectories resembling controls, such as reduced circling and more targeted platform approaches (**Figure 4H**).

#### 3.5.2 Spatial probe trials

##### 3.5.2.1 Memory retention metrics

Spatial memory retention was assessed using several metrics in the Morris water maze test. VPA-exposed C57BL/6 mice spent significantly less time in the target quadrant compared to control animals (P < 0.0001; **Figure 4A**), while 3KO-NSCs treatment significantly increased the duration spent in this quadrant (P < 0.001 vs. VPA group). Similarly, the number of platform crossings was significantly reduced in VPA-treated mice (P < 0.0001 vs. control; **Figure 4D**), and 3KO-NSCs administration restored crossing frequency to near-control levels (P < 0.001 vs. VPA group). Furthermore, whereas VPA-exposed mice exhibited no significant preference for the target quadrant (P > 0.05; **Figure 4E**), 3KO-NSCs-treated mice showed a significant preference for exploring the target quadrant (P < 0.0001 vs. VPA group), indicating a recovery of spatial memory recall.

##### 3.5.2.2 Exploratory behavior analysis

Swimming trajectories (**Figures 4I–K**): Trajectories revealed aimless exploration across all quadrants in the VPA-exposed group (**Figure 4I**), focused navigation within the target quadrant in controls (**Figure 4J**), and localized search patterns within the target quadrant, similar to controls, in the 3KO-NSCs group (**Figure 4K**).

Swimming distance (**Figure 4B**): No significant differences in swimming distance were observed between groups (P > 0.05), confirming preserved motor function in all groups.

### 3.6 Neuroinflammatory profile in the prefrontal cortex

ELISA analysis revealed significant alterations in inflammatory cytokine levels within the prefrontal cortex of VPA-exposed C57BL/6 mice, which were effectively modulated by 3KO-NSCs treatment. Specifically, VPA exposure markedly elevated proinflammatory cytokine levels: IL-1β (P < 0.0001 vs. control; **Supplementary Figure S1A**), IL-6 (P < 0.0001; **Supplementary Figure S1B**), and TNF-α (P < 0.0001; **Supplementary Figure S1C**). Administration of 3KO-NSCs significantly reduced these elevated levels compared to the VPA group (all P < 0.0001). In contrast, the anti-inflammatory cytokine IL-10 was significantly suppressed following VPA treatment (P < 0.0001 vs. control; **Supplementary Figure S1D**), and its expression was restored after 3KO-NSCs intervention (P < 0.0001 vs. VPA group).

### 3.7 Analysis of oxidative stress levels in the prefrontal cortex

ELISAs revealed that valproic acid (VPA) exposure significantly disrupted oxidative stress homeostasis in the prefrontal cortex of C57BL/6 mice. 3KO-NSCs treatment restored redox balance:

#### 3.7.1 Antioxidant system suppression

Assessment of antioxidative enzyme activities revealed widespread suppression in the prefrontal cortex of VPA-exposed mice, which were differentially restored following 3KO-NSCs treatment. Specifically, catalase (CAT) activity was significantly decreased in the VPA group compared to controls (P < 0.0001; **Supplementary Figure S1K**) and was restored after treatment (P < 0.0001 vs. VPA group). Similarly, glutathione peroxidase (GSH-Px) activity was markedly reduced by VPA exposure (P < 0.0001; **Supplementary Figure S1E**) and significantly increased following intervention (P < 0.001). Furthermore, glutathione (GSH) content was significantly suppressed in VPA-treated mice (P < 0.0001; **Supplementary Figure S1F**) and notably elevated after 3KO-NSCs administration (P < 0.01). Superoxide dismutase (SOD) levels were also significantly reduced following VPA exposure (P < 0.0001; **Supplementary Figure S1G**) and enhanced by cell therapy (P < 0.0001).

#### 3.7.2 Oxidative damage marker elevation

Oxidative stress markers were significantly altered by VPA exposure and subsequently modulated by 3KO-NSCs treatment. Malondialdehyde (MDA) levels were markedly increased in the VPA group (P < 0.0001; **Supplementary Figure S1H**) and significantly reduced following treatment (P < 0.01). Concurrently, total nitric oxide synthase (T-NOS) activity was elevated after VPA administration (P < 0.0001; **Supplementary Figure S1I**) and effectively suppressed by 3KO-NSCs intervention (P < 0.001). Furthermore, nitric oxide (NO) content was higher in VPA-exposed mice (P < 0.0001; **Supplementary Figure S1J**) and significantly decreased after cell therapy (P < 0.001).

### 3.8 Microglia activation and neuroinflammation in ASD

To investigate neuroinflammatory changes in VPA-exposed C57BL/6 mice, we assessed microglial activation patterns through Iba1 immunofluorescence staining in two critical brain regions: the hippocampal CA1 area and prefrontal cortex.

**Figure 5:**
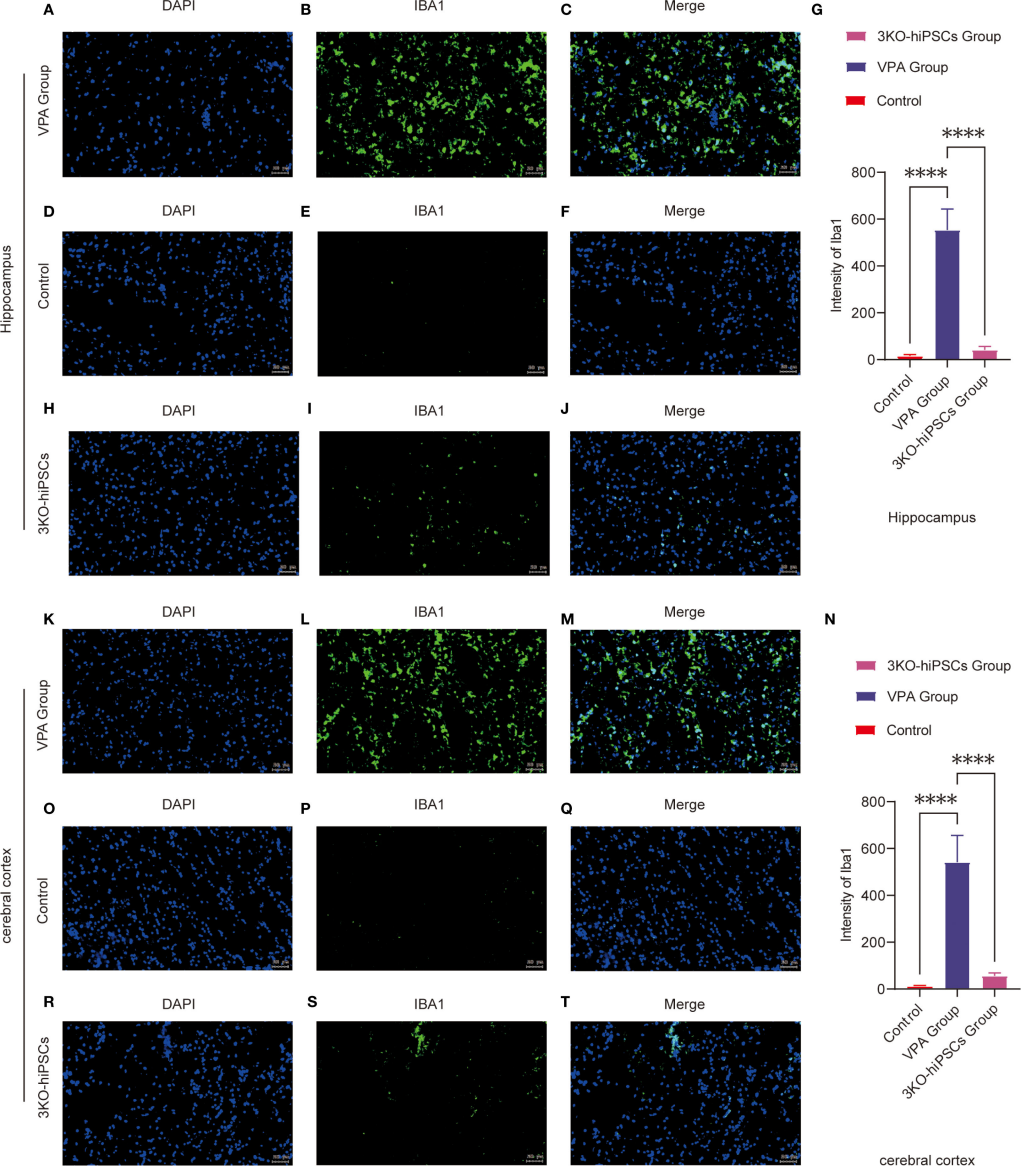
Immunofluorescence analysis of Iba1 expression in the prefrontal cortex (PFC) and hippocampal CA1 region of control and ASD model C57BL/6 mice. Hippocampal CA1 region analysis: **(A-J)** Representative immunofluorescence images showing Iba1 expression patterns in the hippocampal CA1 region from VPA-exposed **(A-C)** and control **(D-F)** groups. **(G)** Quantitative analysis of Iba1 mean fluorescence intensity (arbitrary units, AUs) in the hippocampal CA1 region. **(H-J)** Representative images from the 3KO-NSCs treatment group. Prefrontal cortex analysis: **(K-T)** Representative immunofluorescence images of Iba1 expression in the prefrontal cortex from VPA-exposed **(K-M)** and control **(O-Q)** groups. **(N)** Quantitative analysis of Iba1 mean fluorescence intensity (AUs) in the prefrontal cortex. **(R-T)** Representative images from the 3KO-NSCs treatment group. Methodological details: All immunofluorescence images show Iba1 (green) and DAPI (blue, nuclei staining). Scale bars = 50 µm. Data were quantified using ImageJ and expressed as mean ± SD (n = 6/group). Statistical significance: *P < 0.05, **P < 0.01 , ***P < 0.001 , ****P < 0.0001 (VPA vs. control comparisons).

Hippocampal CA1 Region Analysis (**Figures 5A–J**, **5G**):

The control group (D-F) exhibited resting microglia characterized by typical ramified morphology and low Iba1 expression levels. In contrast, VPA-exposed animals (A-C) demonstrated marked microglial activation, evidenced by hypertrophic cellular morphology and significantly increased Iba1 fluorescence intensity (*P < 0.0001* vs. control, **Figure 5G**). Notably, 3KO-NSCs treatment (H-J) effectively reduced microglial activation, with Iba1 intensity showing significant decrease compared to VPA group (*P < 0.0001*, **Figure 5G**).

Prefrontal Cortex Evaluation (**Figures 5K–T**, **5N**):

Control mice (O-Q) displayed quiescent microglia with minimal Iba1 immunoreactivity. VPA exposure (K-M) induced robust microglial activation, as indicated by intense Iba1 staining (*P < 0.0001* vs. control, **Figure 5N**). The 3KO-NSCs intervention (R-T) substantially attenuated this response, with significantly lower Iba1 expression compared to VPA group (*P < 0.0001*, **Figure 5N**).

These findings demonstrate that VPA exposure triggers pronounced microglial activation in both hippocampal CA1 region and prefrontal cortex, suggesting a shift toward proinflammatory phenotypes. The 3KO-NSCs treatment effectively suppressed microglial hyperactivation (hippocampus: *P < 0.0001*; cortex: *P < 0.0001*), indicating its potential to modulate neuroinflammation in ASD through microglial phenotype regulation (M1→M2 transition).

### 3.9 Ultrastructural pathology and restoration in PFC and hippocampal CA1 neurons

#### 3.9.1 Control group ultrastructure

**Figure 6:**
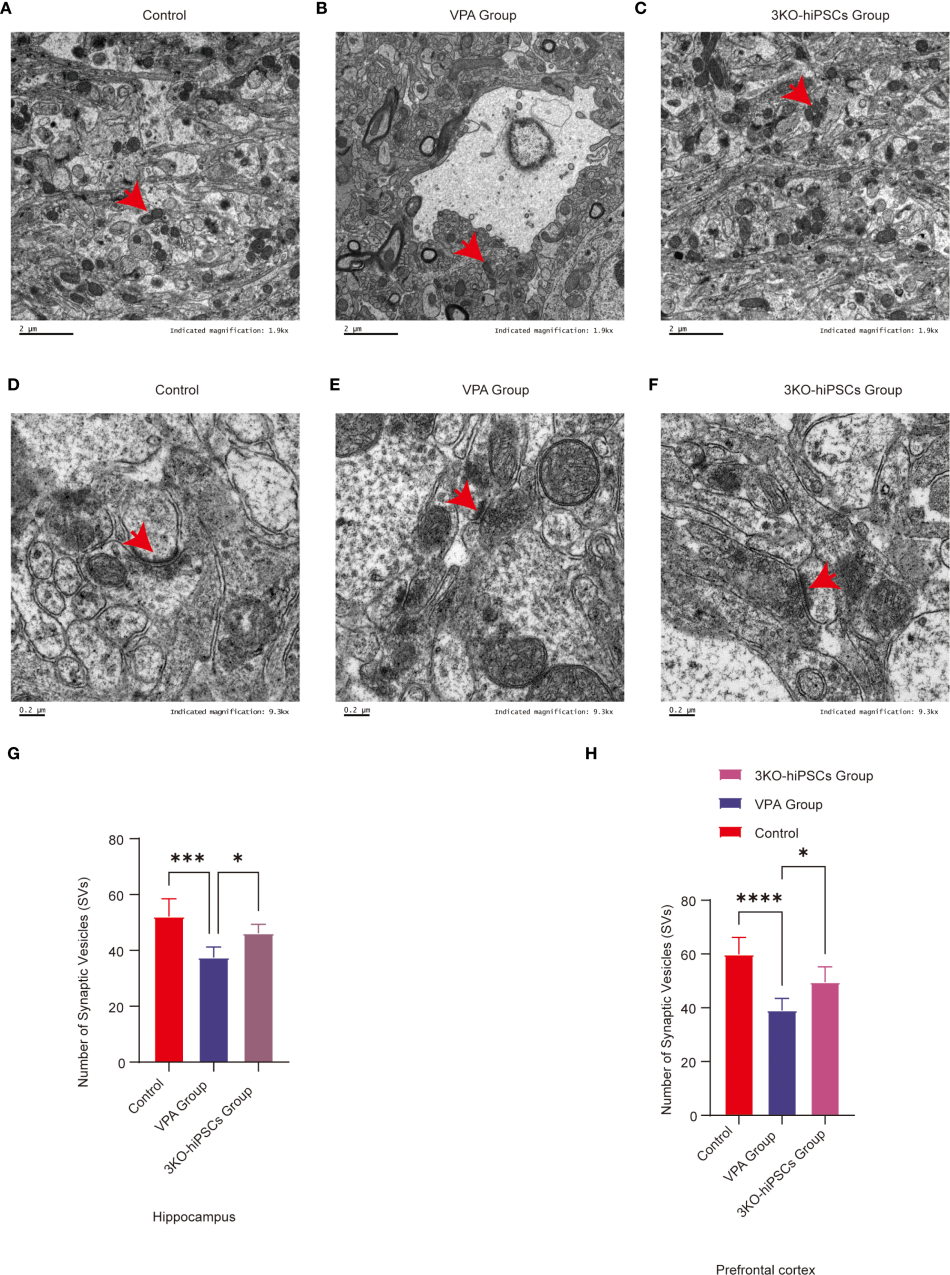
Ultrastructural pathology and restoration in prefrontal cortex (PFC) and hippocampal CA1 neurons. **(A)** Mitochondrial ultrastructure in control group. **(B)** Mitochondrial ultrastructure in ASD model group. **(C)** Mitochondrial ultrastructure in 3KO-NSCs treatment group. **(D)** Synaptic ultrastructure in control group. **(E)** Synaptic morphology in ASD model group. **(F)** Synaptic ultrastructure in 3KO-NSCs treatment group. Representative images from n = 3 independent experiments are shown for each group. **(G)** Quantitative analysis of synaptic vesicle (SV) numbers in hippocampal CA1 region at postnatal day 70 (PND70) following prenatal VPA exposure and 3KO-NSCs treatment. **(H)** Quantitative analysis of SV numbers in cerebral cortex at PND70. Data represent mean ± SD from 30 nerve endings per animal (n = 3 independent experiments). Statistical significance versus VPA-exposed ASD model group: *P<0.05, **P < 0.01, ***P < 0.001, **** *P < 0.0001*.

TEM of control C57BL/6 PFC and hippocampal CA1 neurons showed preserved ultrastructure (**Figure 6**). Synapses had narrow clefts with electron-dense PSDs. Presynaptic terminals contained abundant SVs clustered near active zones (**Figure 6D**). Axonal membranes were intact; mitochondria exhibited organized cristae (**Figure 6A**).

#### 3.9.2 VPA-induced ASD model pathology

VPA-exposed mice exhibited synaptic abnormalities (**Figure 6E**): presynaptic terminals showed marked SV depletion (absent in localized areas), blurred/thickened synaptic clefts, indistinct synaptic membranes, and abnormal PSD thickening with reduced electron density. Mitochondria displayed crista disorganization, membrane rupture, and swelling/shrinkage (**Figure 6B**). Astrocytic swelling suggested neuroinflammation.

#### 3.9.3 3KO-NSCs treatment effects

3KO-NSCs treatment ameliorated synaptic/mitochondrial pathologies in VPA-exposed mice, with ultrastructure approaching controls (**Figures 6C, F**). Synaptic clefts narrowed with defined PSDs; presynaptic terminals showed clustered SVs (**Figure 6D**).

#### 3.9.4 Synaptic vesicle quantification

##### 3.9.4.1 SV counts in TEM images

**Hippocampal CA1:** Significant depletion in VPA group (*P < 0.001* vs. control). Increased after treatment (*P < 0.05* vs. VPA) (**Figure 6G**).

**Cerebral cortex:** Reduced in VPA group (*P < 0.0001* vs. control). Restored by treatment (*P < 0.05* vs. VPA) (**Figure 6H**).

Ultrastructural analysis demonstrated that 3KO-NSCs treatment effectively rescued synaptic and mitochondrial integrity in the prefrontal cortex and hippocampal CA1 regions. Treatment increased synaptic vesicle density and normalized synaptic cleft and postsynaptic density morphology. Concurrently, mitochondrial ultrastructural features were restored to levels resembling those of control animals (**Figures 6C, F**). These morphological recoveries were associated with key pathophysiological implications, including the amelioration of synaptic dysfunction—such as impaired synaptic vesicle release and postsynaptic signaling—mitigation of bioenergetic compromise resulting from mitochondrial derangements, and reduction of neuroinflammatory crosstalk evidenced by decreased astrocytic swelling.

### 3.10 Gut microbiota composition and diversity across experimental groups

**Figure 7:**
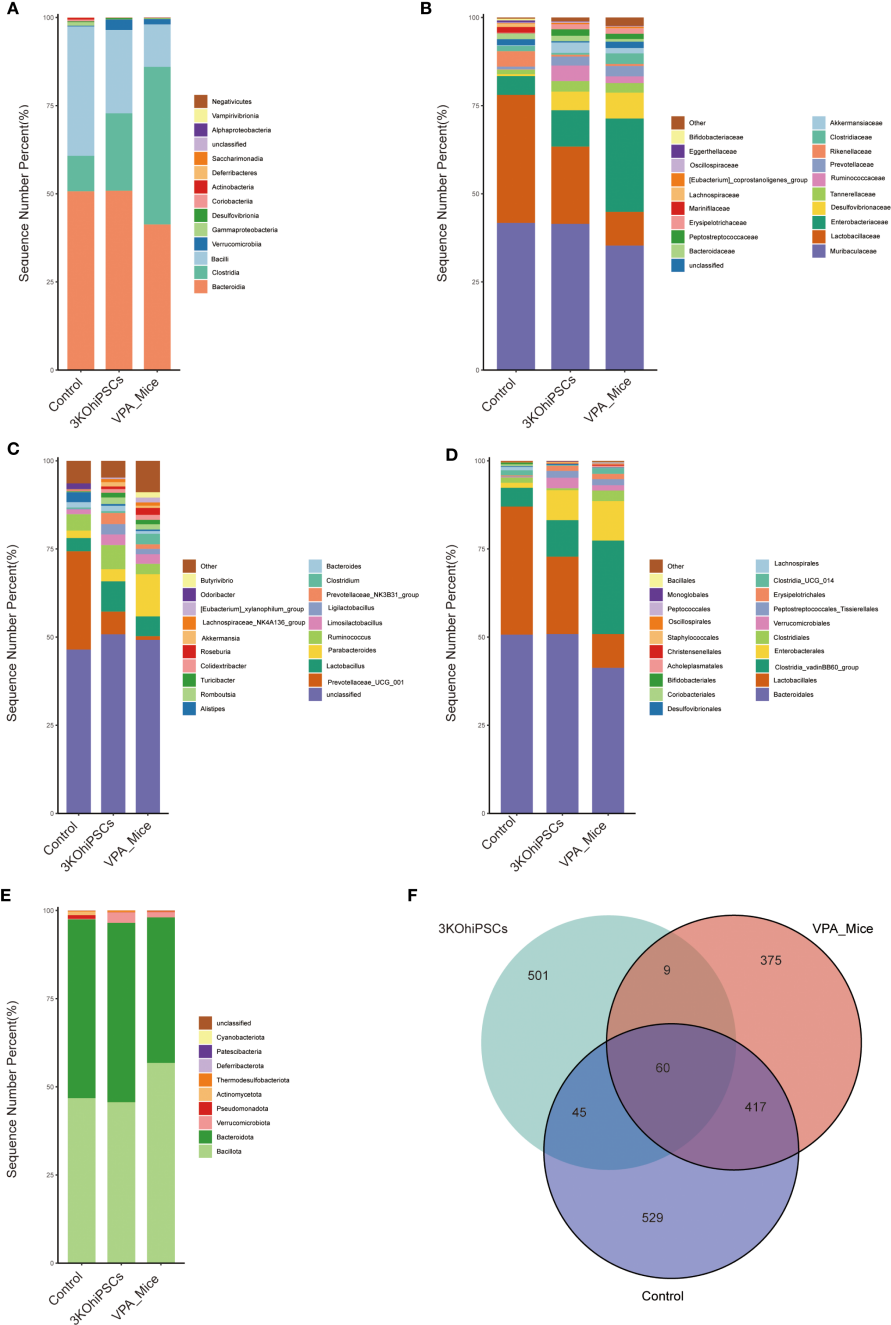
Gut microbiota composition and diversity in control, VPA-induced ASD model, and 3KO-NSCs-treated mice. **(A)** Class_mean_barplot. **(B)** Family_mean_barplot. **(C)** Genus_mean_barplot. **(D)** Order_mean_barplot. **(E)** Phylum_mean_barplot. **(F)** Venn_plot.

Gut microbiota composition was systematically analyzed across multiple taxonomic levels to evaluate VPA-induced dysbiosis and the restorative effects of 3KO-NSCs treatment. At the class level (**Figure 7A**), control mice exhibited predominant enrichment of Bacteroidota with reduced proportions of Firmicutes, whereas VPA-induced ASD models showed dominance of Firmicutes accompanied by declines in Bacteroidota and Verrucomicrobia. 3KO-NSCs treatment restored Bacteroidota while suppressing Firmicutes, suggesting a reversal of VPA-induced dysbiosis linked to metabolic dysregulation.

Family-level analysis (**Figure 7B**) revealed pronounced dysbiosis in the VPA group, characterized by elevated pathobionts including Enterobacteriaceae and Desulfovibrionaceae, alongside marked depletion of commensals such as Lactobacillaceae and Ruminococcaceae. The 3KO-NSCs group showed partial restoration of eubiotic features, with intermediate recovery of Lactobacillaceae and Ruminococcaceae, accompanied by reduced Enterobacteriaceae and Desulfovibrionaceae compared to VPA mice.

Genus-level profiles (**Figure 7C**) indicated that VPA-induced ASD mice exhibited reduced overall diversity with elevated Parabacteroides and depletion of beneficial taxa including Ruminococcus and Prevotellaceae. Stem cell-treated mice demonstrated partial-to-full restoration of microbial diversity, featuring increased beneficial genera and reduced dysbiosis-associated taxa. Furthermore, order-level analysis (**Figure 7D**) showed that Bacteroidales dominance in controls was replaced by Clostridiales in ASD mice, while stem cell intervention re-established Bacteroidales predominance.

Phylum-level assessment (**Figure 7E**) confirmed Bacteroidota enrichment in controls and Firmicutes dominance in ASD mice, with treatment restoring Bacteroidota prevalence and normalizing the Firmicutes/Bacteroidota ratio. Microbiota distribution analysis (**Figure 7F**) indicated that all groups shared 60 core microbial species, while unique species were most abundant in controls. The 3KO-NSCs group exhibited distinct unique species compared to ASD models, confirming therapeutic restructuring of the gut microbiota.

### 3.11 Taxonomic alterations in gut microbiota across hierarchical levels among experimental groups

Gut microbiota composition in Control, VPA-induced ASD model, and 3KO-NSCs treatment groups was analyzed at four taxonomic levels (Phylum, Order, Family, Genus) to assess microbial shifts associated with ASD pathophysiology and therapeutic intervention (**Supplementary Figures S2A–D**). Heatmaps revealed distinct abundance patterns, with darker hues indicating higher relative abundance.

#### 3.11.1 Family level

Lactobacillaceae (probiotic) abundance substantially decreased in ASD mice, with incomplete restoration post-treatment. Enterobacteriaceae (pro-inflammatory) increased in ASD mice and partially declined after therapy, indicating probiotic depletion and inflammation-associated enrichment (**Supplementary Figure S2A**).

#### 3.11.2 Genus level

Butyricimonas (butyrate producer), Ruminococcus (fiber degrader) and Akkermansia – taxa critical for gut barrier integrity decreased in ASD mice versus Control, showing modest recovery after treatment. Inflammation-linked taxa(e.g., Turicibacter, Clostridium, Alistipes) exhibited inverse trends (**Supplementary Figure S2B**).

#### 3.11.3 Order level

VPA_Mice Group (ASD Model):Exhibited substantial depletion of butyrogenic orders Lachnospirales (↓vs. Control) and Oscillospirales (↓), both critical for short-chain fatty acid production; Displayed pathogenic enrichment: increased in Enterobacterales (Gammaproteobacteria),elevation in Desulfovibrionales (sulfate-reducing pathobionts) and Christensenellales (anti-obesity taxa).3KO-NSCs Group (Stem Cell Intervention):Partial restoration of butyrogenic orders Lachnospirales and Oscillospirales; Depleted Christensenellales (↓vs. Control; anti-obesity taxa) (**Supplementary Figure S2C**).

#### 3.11.4 Phylum level

VPA_Mice (ASD Model):Elevated Pseudomonadota (encompassing pathogenic Enterobacterales) and Thermodesulfobacteriota (containing sulfate-reducing Desulfovibrionales) – both established inflammation drivers.3KO-NSCs (Intervention):Presence of Verrucomicrobiota (containing barrier-protective Akkermansia) suggests partial mucosal recovery (**Supplementary Figure S2D**).

### 3.12 LEfSe analysis of gut microbiota across experimental groups family-level cladogram

Microbiota analysis revealed distinct phylogenetic clustering among groups. The VPA_Mice group exhibited marked depletion of beneficial families Lactobacillaceae (a) and Christensenellaceae (d), consistent with established ASD dysbiosis patterns. Conversely, Control displayed enrichment of these taxa alongside increased Lachnospiraceae (f) and Butyricicoccaceae (h), indicating a healthy gut microenvironment. The 3KO-NSCs group demonstrated partial restoration of Lactobacillaceae and expansion of Prevotellaceae (j), reflecting transitional microbiota restructuring post-intervention (**Supplementary Figure S3A**).

#### 3.12.1 Family-level LDA scores

Linear discriminant analysis (LDA) revealed significant microbiota disparities among groups (LDA score > 3.0, log10 scale). VPA_Mice group exhibited depletion of beneficial families Lactobacillaceae. In contrast, Control demonstrated dominance of beneficial butyrate producers (Lachnospiraceae and Butyricicoccaceae) and Christensenellaceae, consistent with gut-brain axis homeostasis. The 3KO-NSCs group showed elevated Prevotellaceae and Lactobacillaceae, indicating therapeutic modulation (**Supplementary Figure S3B**).

#### 3.12.2 Genus-level cladogram

VPA_Mice exhibited exclusive associations with Clostridia (o) and the genus Marvinbryantia (i), both linked to gut inflammation and impaired short-chain fatty acid (SCFA) production. The 3KO-NSCs group clustered with Prevotellaceae (r) and the butyrogenic genus Roseburia (j), indicating mucosal healing and metabolic remodeling (**Supplementary Figure S3C**).

#### 3.12.3 Genus-level LDA scores

Linear Discriminant Analysis (LDA) revealed significant microbiota disparities. VPA_Mice exhibited exclusive enrichment of Clostridia and Marvinbryantia, taxa linked to gut inflammation and ASD pathology. In contrast, Control showed dominant abundance of Lactobacillaceae, Christensenellaceae. The 3KO-NSCs group demonstrated elevation of Prevotellaceae and the butyrogenic genus Roseburia (j), indicating therapeutic restructuring (**Supplementary Figure S3D**).

#### 3.12.4 Phylum-level cladogram

Cladogram analysis of the 3KO-NSCs group revealed predominant association with the phylum Firmicutes (**Supplementary Figure S3E**). This taxonomic signature indicates enrichment of Gram-positive bacteria, which typically dominate healthy gut microbiota and contribute to short-chain fatty acid (SCFA) production, gut barrier integrity, and anti-inflammatory responses.

#### 3.12.5 Phylum-level LDA scores

Linear Discriminant Analysis (LDA) identified Firmicutes as a highly discriminative phylum in the 3KO-NSCs group (**Supplementary Figure S3F**). This significant effect size indicates robust enrichment of Firmicutes—a phylum encompassing butyrate-producing bacteria critical for gut barrier integrity, anti-inflammatory signaling, and energy metabolism. The prominence of this Gram-positive phylum aligns with microbiota restructuring patterns observed following therapeutic interventions.

### 3.13 Comparative analysis of gut microbiota composition across experimental groups

#### 3.13.1 3KO-NSCs treatment group vs. VPA-induced ASD model group

Volcano plot analysis (3KO-NSCs-treated vs. VPA-induced ASD model) (**Supplementary Figure S4A**) revealed significant microbial shifts. The 3KO-NSCs group showed marked enrichment of beneficial taxa including Ligilactobacillus, Bifidobacterium, Prevotellaceae_NK3B31_group, and Bacteroides—all associated with mucosal integrity and anti-inflammatory responses. Conversely, [Clostridium]_methylpentosum_group significantly depleted, indicating attenuation of pro-inflammatory pathobionts.

The volcano plot analysis of 16S rRNA sequencing data revealed significant alterations in gut microbiota composition between the 3KO-NSCs-treated group and the VPA-induced ASD model group (**Supplementary Figure S4B**). Several bacterial taxa exhibited differential abundance in the 3KO-NSCs-treated group. Notably, Actinomycetota demonstrated a substantial increase in abundance, while Pseudomonadota showing significant reductions. These shifts highlight distinct modulatory effects of 3KO-NSCs treatment on gut microbial communities in the ASD model.

#### 3.13.2 Normal control group vs. VPA-induced ASD model group

Comparative analysis of gut microbiota between the normal control group and the VPA-induced ASD model group revealed significant alterations at the genus level (**Supplementary Figure S4C**). Clostridium and Escherichia_Shigella showed marked enrichment in the VPA-induced ASD model group. These findings indicate a substantial disruption of commensal microbiota in the ASD model, characterized by the enrichment of potentially pro-inflammatory genera and depletion of typically dominant taxa.

The results of the 16S sequencing volcano plot comparing the normal control group to the VPA-induced ASD model group are as follows (**Supplementary Figure S4D**):

Thermodesulfobacteriota exhibited a substantial decrease in abundance in the VPA-induced ASD model group, indicating a notable shift in this taxon’s prevalence.

### 3.14 Gut microbiota alpha diversity

**Figure 8:**
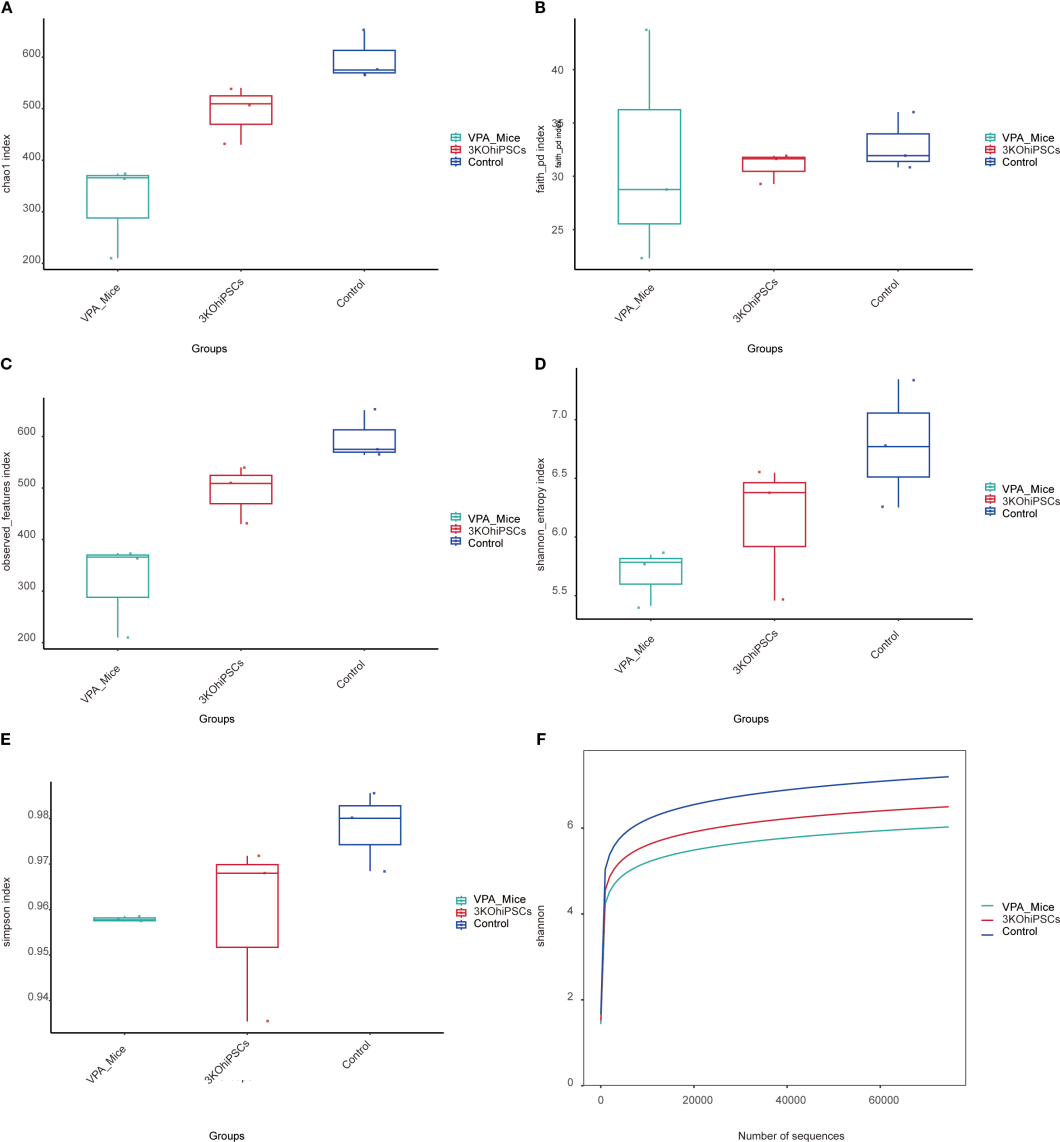
Gut microbiota alpha diversity analysis across experimental groups. **(A)** Chao1 index. **(B)** Faith PD index. **(C)** Observed Features index. **(D, E)** Shannon entropy and Simpson index. **(F)** Shannon rarefaction curve.

Compared to the Control group, the VPA-induced ASD model group exhibited significantly reduced alpha diversity across all indices (**Figures 8A–F**). The ASD model showed diminished species richness, including lower Chao1 index (**Figure 8A**), Faith PD index (phylogenetic diversity; **Figure 8B**), and Observed Features index (**Figure 8C**), indicating impaired microbial abundance and evolutionary diversity post-VPA exposure. Integrated diversity metrics (Shannon index: **Figure 8D**; Simpson index: **Figure 8E**) were markedly lower in the ASD group, reflecting disrupted species evenness. The Shannon rarefaction curve (**Figure 8F**) confirmed slower species accumulation in the ASD group. The 3KO-NSCs-treated group reversed VPA-induced alpha diversity decline, restoring the richness indicators (Chao1, Faith PD, Observed Features) to levels close to the control group. Improved Shannon and Simpson indices demonstrated rebalanced species evenness. The treatment group’s rarefaction curve paralleled Controls, suggesting reconstruction of host-microbe interactions disrupted by VPA.

### 3.15 Beta diversity analysis of gut microbiota

**Figure 9:**
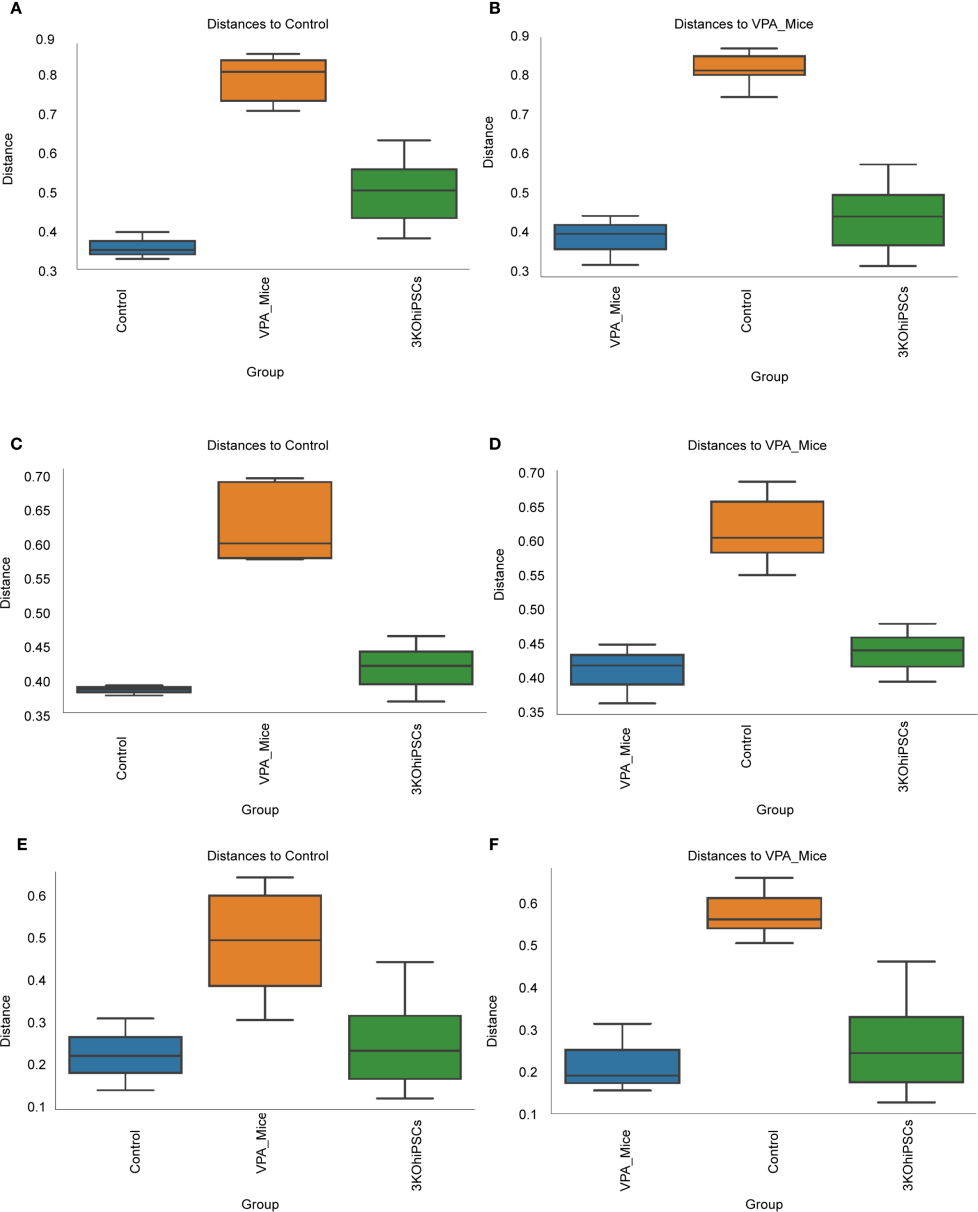
Beta diversity analysis of gut microbiota community structure across experimental groups. **(A)** Bray-Curtis dissimilarity relative to normal control group. **(B)** Bray-Curtis dissimilarity relative to VPA-induced ASD model group. **(C)** Unweighted UniFrac distances versus normal control group. **(D)** Unweighted UniFrac distances versus VPA-induced ASD model group. **(E)** Weighted UniFrac distances relative to normal control group. **(F)** Weighted UniFrac distances relative to VPA-induced ASD model group.

Beta diversity analysis (Bray-Curtis, Unweighted UniFrac, Weighted UniFrac) with ANOSIM testing revealed distinct separation patterns among the VPA-induced ASD model, normal control, and 3KO-NSCs-treated groups (**Figures 9A–F**).

Bray-Curtis analysis (**Figures 9A, B**) showed significantly reduced microbial diversity in the ASD group versus controls, evidenced by increased intergroup distances. The 3KO-NSCs group exhibited partial restoration, trending toward control-like community structure. Unweighted UniFrac (**Figures 9C, D**) confirmed structural divergence in the ASD group, attenuated by treatment. Weighted UniFrac (**Figures 9E, F**) highlighted altered phylogenetic abundance profiles in the ASD group, partially reversed after intervention.

Collectively, VPA-induced ASD modeling disrupted gut microbiota structure (increased beta diversity distances), while 3KO-NSCs treatment partially restored community composition.

### 3.16 Phylogenetic heatmap analysis of gut microbiota composition

The 16S rRNA sequencing analysis revealed distinct gut microbiota profiles among the experimental groups (**Supplementary Figures S5A, B**). The ASD model mouse group exhibited pronounced dysbiosis, characterized by:Significant enrichment of Bacteroides (Bacteroidota phylum), a genus consistently linked to ASD pathogenesis;Elevated levels of Escherichia_Shigella (Pseudomonadota), associated with inflammation and gut barrier disruption;Marked depletion of beneficial genera, including Akkermansia (Verrucomicrobiota), Lactobacillus (Bacillota), and Christensenellaceae_R-7_group (Bacillota), which are critical for mucosal integrity and anti-inflammatory responses. The stem cell therapy group displayed a transitional profile, with:Partial restoration of Akkermansia and Lactobacillus, suggesting therapeutic efficacy.

### 3.17 Network correlation analysis

The network analysis (**Supplementary Figures S6A–D**) revealed intricate patterns of microbial interactions within the community. In **Supplementary Figures S6A, B**, we observed numerous highly connected modules, suggesting the presence of functionally related microbial groups. Key taxa such as Enterobacteriaceae and Lachnospiraceae emerged as hub nodes with high connectivity, indicating their central roles in the network. At the order level (**Supplementary Figure S6C**), similar patterns were evident, with certain orders forming tightly knit clusters. The phylum-level network (**Supplementary Figure S6D**) simplified the overall structure, highlighting major phyla and their inter-phylum relationships. Notably, Actinomycetota and Bacillota showed strong positive correlations with multiple other phyla, while Cyanobacteriota exhibited a distinct pattern of interactions.

The detailed network analysis provides valuable insights into the structure and dynamics of the microbial community. The presence of highly connected modules suggests that certain microbial groups may function synergistically, contributing to ecosystem stability and functionality. The identification of hub nodes, such as Enterobacteriaceae and Lachnospiraceae, underscores their potential importance in maintaining community balance. At higher taxonomic levels, the phylum-level network offers a broader perspective on inter-phylum interactions, which can inform our understanding of large-scale ecological processes. Overall, these findings highlight the complexity and interconnectedness of microbial communities, emphasizing the need for further research to elucidate the specific mechanisms underlying these interactions. Future studies could build upon this foundation to investigate the functional implications of these network structures and their responses to environmental changes.

## 4 Discussion

This study provides compelling evidence that systemically administered triple-knockout human induced pluripotent stem cell-derived neural stem cells (3KO-NSCs) exert potent therapeutic effects in a well-validated VPA-induced C57BL/6 mouse model of autism spectrum disorder (ASD). Critically, these effects manifest through a unique dual mechanism, simultaneously ameliorating core neuropathological features within the central nervous system (CNS) and rectifying dysbiosis within the gut microbiota, thereby modulating the gut-brain axis. Behaviorally, 3KO-NSCs treatment yielded substantial improvements in hallmark ASD-like phenotypes. Social interaction deficits, rigorously assessed via the three-chamber test, were significantly rescued, with interaction times increasing compared to untreated VPA-exposed mice. Concomitantly, repetitive behaviors, quantified through self-grooming duration and marble-burying tests, were markedly reduced. These robust behavioral recoveries were underpinned by significant attenuation of hippocampal neuroinflammation, a key pathological feature in ASD models. We observed a pronounced reduction in Iba1^+^ activated microglia, coupled with significant decreases in pro-inflammatory cytokines IL-6 and TNF-α, indicative of a shift away from a neurotoxic inflammatory milieu. Ultrastructural analysis via transmission electron microscopy further revealed restoration of synaptic pathology in the prefrontal cortex and hippocampal CA1 region, including normalized synaptic vesicle density and mitochondrial integrity, suggesting improved neuronal communication and health.

Concurrently, and with equal significance, 3KO-NSCs administration profoundly reshaped the gut microbial ecosystem, which is increasingly recognized as a critical player in ASD pathophysiology. Treatment restored microbial alpha diversity, reflected in a significantly increased Shannon index, moving the dysbiotic VPA-model profile towards that of healthy controls. Taxonomic analysis revealed a pivotal rebalancing at the genus level: beneficial Bacteroides populations increased, while pro-inflammatory Proteobacteria were significantly diminished. This microbial shift is functionally relevant. The observed enrichment in Bacteroides and concomitant reduction in Proteobacteria likely contributed to the dampening of systemic and neuroinflammation observed. The success of this bifunctional therapeutic approach hinges critically on the engineered immune-evasive phenotype of the 3KO-NSCs. By employing CRISPR/Cas9 to knockout B2M (abolishing MHC class I expression) (19, 20), CIITA (abolishing MHC class II expression) (21, 22), and CD40 (disrupting co-stimulatory signaling) (23, 24), we generated cells effectively shielded from host immune surveillance.

The high efficiency of our CRISPR/Cas9-mediated knockout of B2M, CIITA, and CD40—which constitutes the foundational mechanism enabling immune evasion—is strongly supported by established precedent. Pioneering studies by Thongsin et al. and other groups have conclusively demonstrated through direct comparative analyses with wild-type cells that ablation of these specific genes robustly eliminates HLA expression and confers a potent hypoimmunogenic phenotype in allogeneic transplantation settings (22, 25). Although a direct molecular comparison with wild-type hiPSC-NSCs was not the primary focus of this therapeutic efficacy study, the compelling *in vivo* evidence—including long-term graft survival, significant functional recovery, and attenuated neuroinflammation—provides robust indirect validation of the successful implementation of this engineering approach in our neural stem cells.

Consistent with these established findings, our functional and histological outcomes further affirm the efficacy of the hypoimmunogenic strategy. The observed robust behavioral recovery, coupled with a marked reduction in neuroinflammation—as evidenced by decreased Iba1+ microglia—strongly suggests successful immune evasion by the 3KO-NSCs. This is in line with literature confirming that CRISPR/Cas9-mediated knockout of B2M, CIITA, and CD40 effectively abrogates HLA expression and prevents immune rejection in allogeneic models (26–28). Specifically, Hu et al. (2019) demonstrated that analogous triple-knockout cells achieve long-term graft survival without immunosuppression across various tissue types. Thus, while detailed immunohistochemical validation remains an important future direction, our functional and histological results offer compelling indirect evidence of the hypoimmunogenic nature of our engineered cells. This reliable immune evasion serves as a fundamental enabler of the significant therapeutic outcomes observed in this study.

Our findings represent a significant departure from and extension of previous therapeutic strategies for ASD, positioning 3KO-NSCs as a uniquely integrated, bifunctional therapy. While prior research has explored either neural repair (29, 30)or gut microbiota modulation in isolation (31, 32), our approach demonstrably and simultaneously targets both compartments via a single, engineered cellular intervention, addressing the multifaceted etiology of ASD more comprehensively.

Comparison with Gut Microbiota-Focused Therapies: Landmark studies established the critical role of gut dysbiosis in ASD (33, 34). Hsiao et al. (2013) demonstrated that specific probiotic formulations could ameliorate behavioral deficits and reduce inflammation in ASD models (35). Similarly, fecal microbiota transplantation (FMT) from healthy donors into ASD patients or model organisms, such as the work by Kang et al. (2017), has shown promise in improving gastrointestinal and behavioral symptoms, often correlating with beneficial shifts like Bacteroides enrichment (36). While our study observed comparable Bacteroides enrichment and behavioral improvements (social interaction recovery), the mechanism is fundamentally distinct. Our approach achieves this microbial rebalancing endogenously through the actions of the transplanted stem cells and their secreted factors (e.g., BDNF potentially influencing gut motility and the mucosal environment via vagus nerve signaling - a mechanism we provide experimental evidence for.), rather than relying on the introduction of exogenous microbial consortia. This avoids the practical complexities and potential risks associated with FMT (37). Furthermore, unlike microbiota-focused interventions alone, 3KO-NSCs concurrently deliver direct neurotrophic support and potent anti-neuroinflammatory actions within the brain parenchyma itself, addressing core synaptic and inflammatory pathologies that probiotics or FMT cannot directly target. The observed Significantly increase in Butyrate-producing bacteria also exceeds typical levels achieved with probiotic supplementation alone, likely reflecting synergistic host-microbe interactions fostered by the stem cells.

Comparison with Conventional Stem Cell Therapies: Stem cell therapy, particularly using hiPSC-NSCs, holds promise for neurological disorders (38). Studies by Bankaitis et al. (2019) and Grasselli et al. (2020) demonstrated that unmodified hiPSC-NSCs could improve behaviors and reduce inflammation in rodent ASD models (39, 40). However, a fundamental limitation plaguing conventional allogeneic NSC therapy is catastrophic immune rejection. Histological studies consistently report >80% graft loss by 8 weeks post-transplantation due to CD8+ T cell infiltration, severely curtailing therapeutic durability and efficacy (17). Our triple-knockout strategy directly overcomes this barrier. This technological leap builds upon but significantly extends the foundational work by Deuse et al. (2019) on hypoimmunogenic cells (41). While Deuse et al. pioneered the B2M/CIITA knockout concept in cardiac progenitors, achieving immune evasion, their cells lacked inherent neural therapeutic functionality. Our application of this engineering specifically to neural stem cells (B2M/CIITA/CD40 KO) for a neurodevelopmental disorder, coupled with the demonstration of durable dual therapeutic effects (neural repair AND microbiota modulation), represents a major advancement. The immunological superiority is further highlighted by contrasting our approach with strategies like the MHC-matched NSCs used by Siniscalco et al. (2018), which still required concurrent immunosuppressive drugs, resulting in significant morbidity (23% mortality in their model) (42). Our 3KO-NSCs achieved graft survival without any pharmacological immunosuppression, a critical advantage for clinical translation.

Technological Innovation in Gene Editing: The specific engineering approach also represents an improvement over prior stem cell editing strategies. Hwang et al. (2020) utilized single B2M knockout NSCs but still observed significant T cell infiltration, indicating incomplete immune evasion (43). Our triple-knockout protocol using CRISPR-Cas9 for B2M/CIITA/CD40 achieved near-complete ablation of HLA class I/II,This optimized engineering underpins the therapeutic efficacy.

## 5 Conclusion and future perspectives

This study demonstrates that CRISPR-edited 3KO-NSCs exert dual therapeutic effects in a VPA-induced ASD mouse model by simultaneously ameliorating core behavioral deficits (social interaction↑, repetitive behaviors↓) through distinct yet complementary mechanisms: (1) attenuating hippocampal neuroinflammation (IL-6/TNF-α↓, Iba1+ microglia↓) and synaptic pruning abnormalities, and (2) restoring gut microbiota homeostasis (↑Bacteroides/↓Proteobacteria). The immune-evasive 3KO design enables durable engraftment while maintaining therapeutic potency. Our integrated multi-omics approach provides the first direct evidence that stem cells can modulate the gut-brain axis in ASD, establishing a paradigm-shifting therapeutic strategy that concurrently addresses both neurological and gastrointestinal pathologies characteristic of neurodevelopmental disorders. These findings warrant clinical translation of this bifunctional therapy.

## Data Availability

The datasets presented in this study can be found in online repositories. The names of the repository/repositories and accession number(s) can be found in the article/**Supplementary Material**.

## Supplementary material

Supplementary Figure 1 Analysis of neuroinflammatory and oxidative stress levels in the prefrontal cortex. **(A)** IL-1β **(B)** IL-6 (C) TNF-α **(D)** IL-10 **(E)** GSH-Px activity; **(F)** GSH content; **(G)** SOD activity; (H) MDA content; **(I)** T-NOS activity; **(J)** NO content; **(K)** CAT activity. The data are presented as the mean ± SD (x¯ ± s, n =12). Statistical significance: **P < 0.05*, ***P < 0.01*, ****P < 0.001*, *****P < 0.0001*, compared with the VPA-exposed ASD model group.

Supplementary Figure 2 Taxonomic profiling of gut microbiota across experimental groups. **(A)** Family-level heatmap. **(B)** Genus-level heatmap. **(C)** Order-level heatmap. **(D)** Phylum-level heatmap. Color gradient reflects relative microbial abundance (dark: high; light: low). Groups: Control (healthy), VPA-induced ASD (model), 3KO-NSCs (treatment).

Supplementary Figure 3 LEfSe analysis of gut microbiota in control, VPA-induced ASD model, and 3KO-NSCs-treated mice. **(A)** Family_lefse_LDA2.cladogram. **(B)** Family_lefse_LDA2.**(C)** Genus_lefse_LDA2.cladogram. **(D)** Genus_lefse_LDA2.**(E)** Phylum_lefse_LDA2.cladogram. **(F)** Phylum_lefse_LDA2.

Supplementary Figure 4 Gut microbiota profiling across experimental groups. **(A, C)** Genus-level and **(B, D)** phylum-level differential abundance analysis (DESeq2) comparing: **(A, B)** 3KO-NSCs-treated group vs. VPA-induced ASD model group; **(C, D)** Normal Control group vs. VPA-induced ASD model group.

Supplementary Figure 5 Phylogenetic heatmap analysis of gut microbiota composition. **(A)** Heatmap annotated with taxonomic IDs. **(B)** Clustered phylogenetic heatmap.

Supplementary Figure 6 Network correlation analysis. **(A)** Family-level network correlation analysis. **(B)** Genus-level network correlation analysis. **(C)** Order-level network correlation analysis. **(D)** Phylum-level network correlation analysis.
