# Supplementary figures and images for: 3KO-NSCs ameliorate behavioral deficits and modulate gut microbiota in a VPA-induced C57BL/6 mouse model of autism
Source: Front Immunol. 2025 Oct 2;16:1680179. doi: 10.3389/fimmu.2025.1680179 (PMC12528095; doi:10.3389/fimmu.2025.1680179)

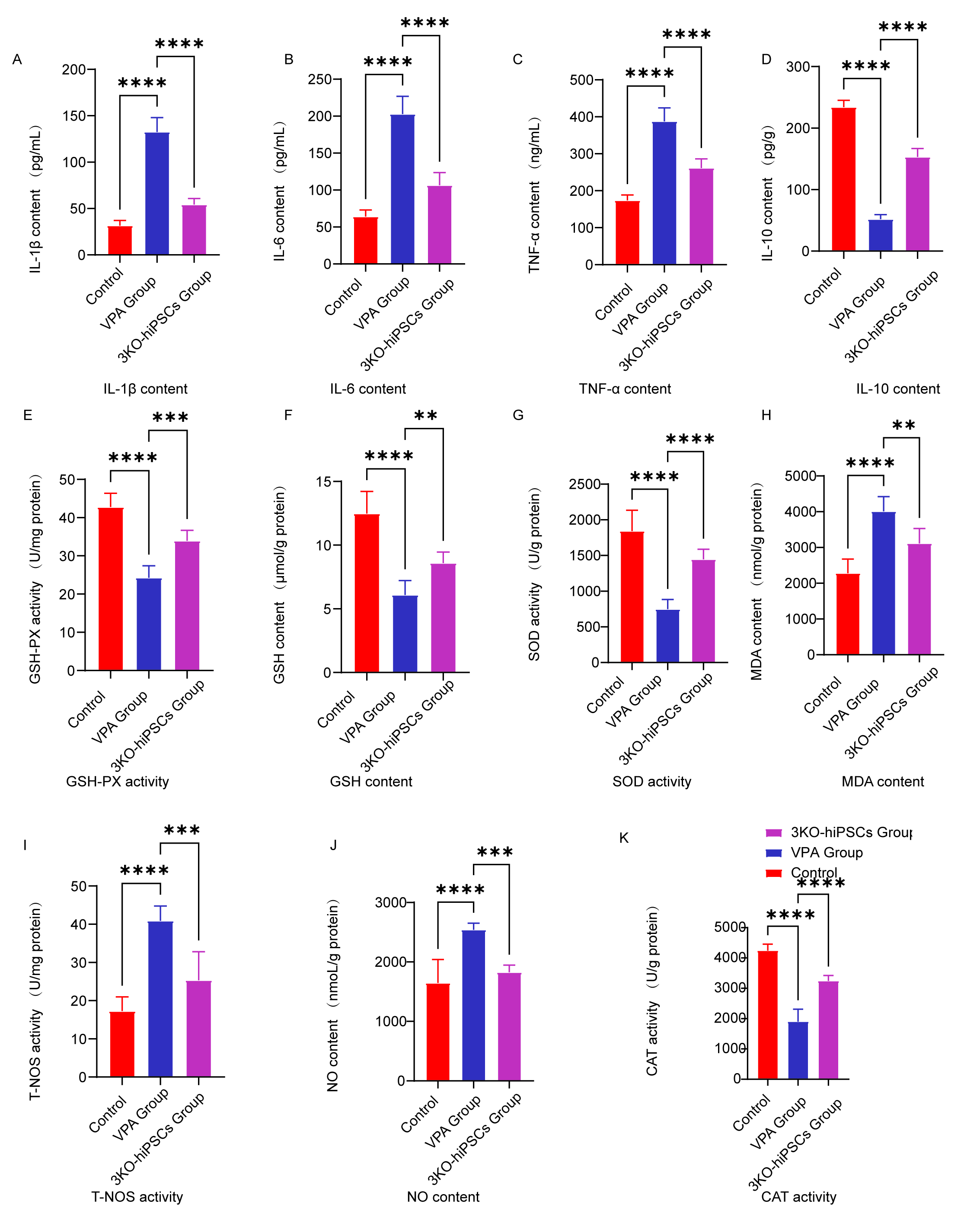

Supplement: Supplementary Figure 1 — Analysis of neuroinflammatory and oxidative stress levels in the prefrontal cortex. (A) IL-1β (B) IL-6 (C) TNF-α (D) IL-10 (E) GSH-Px activity; (F) GSH content; (G) SOD activity; (H) MDA content; (I) T-NOS activity; (J) NO content; (K) CAT activity. The data are presented as the mean ± SD (x¯ ± s, n =12). Statistical significance: *P < 0.05, **P < 0.01, ***P < 0.001, ****P < 0.0001, compared with the VPA-exposed ASD model group. [file Image1.tif]

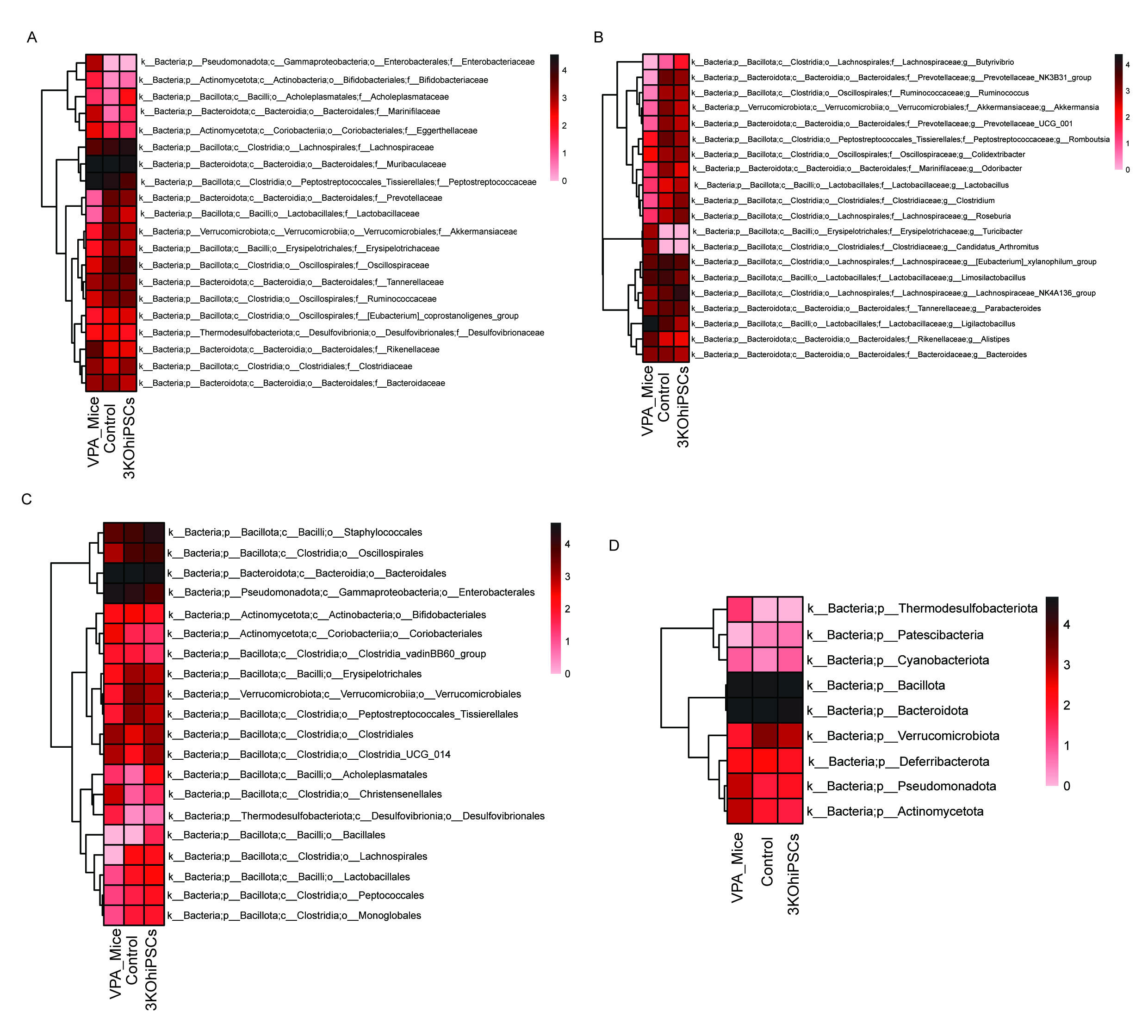

Supplement: Supplementary Figure 2 — Taxonomic profiling of gut microbiota across experimental groups. (A) Family-level heatmap. (B) Genus-level heatmap. (C) Order-level heatmap. (D) Phylum-level heatmap. Color gradient reflects relative microbial abundance (dark: high; light: low). Groups: Control (healthy), VPA-induced ASD (model), 3KO-NSCs (treatment). [file Image2.tif]

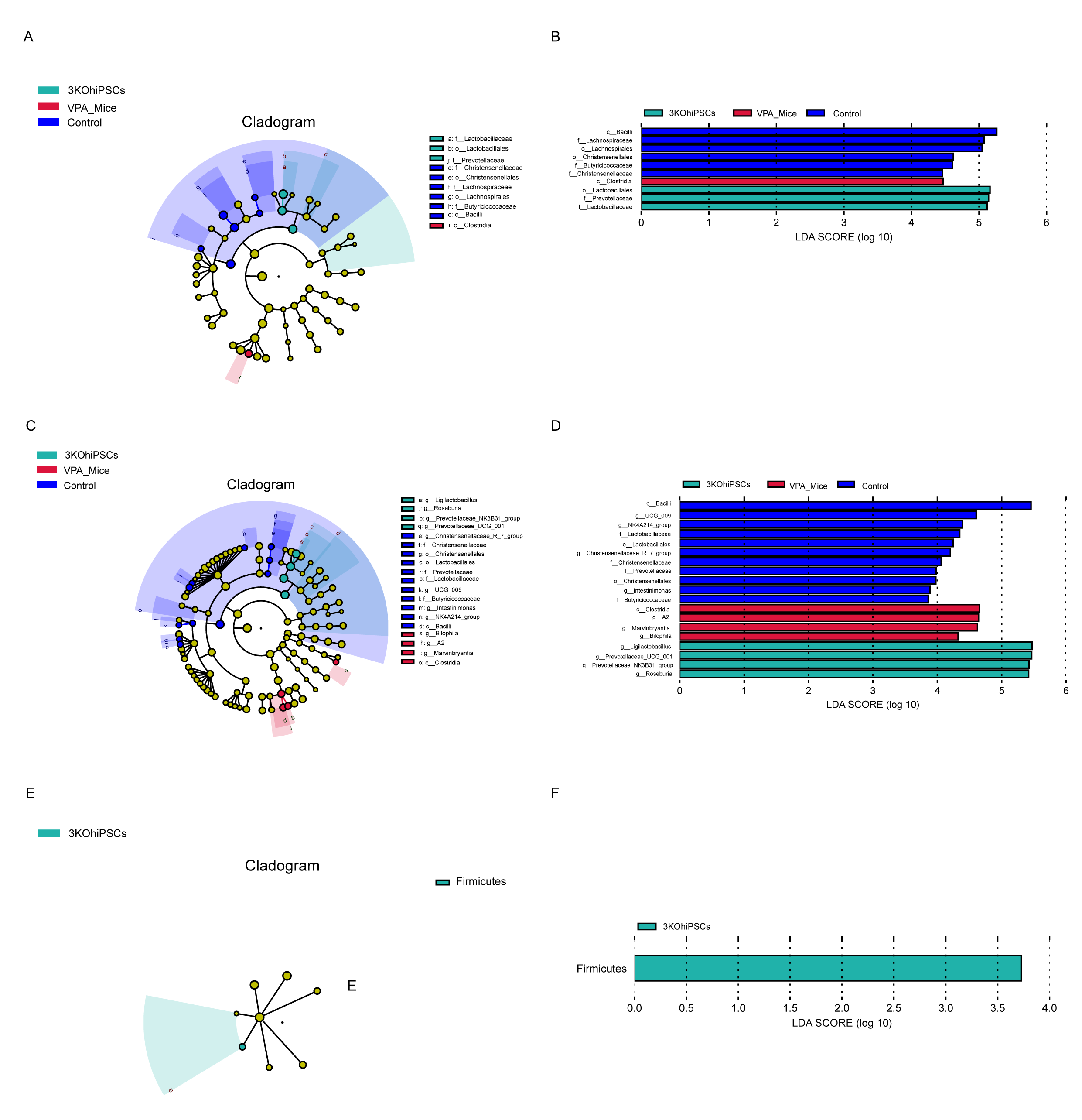

Supplement: Supplementary Figure 3 — LEfSe analysis of gut microbiota in control, VPA-induced ASD model, and 3KO-NSCs-treated mice. (A) Family_lefse_LDA2.cladogram. (B) Family_lefse_LDA2.(C) Genus_lefse_LDA2.cladogram. (D) Genus_lefse_LDA2.(E) Phylum_lefse_LDA2.cladogram. (F) Phylum_lefse_LDA2. [file Image3.tif]

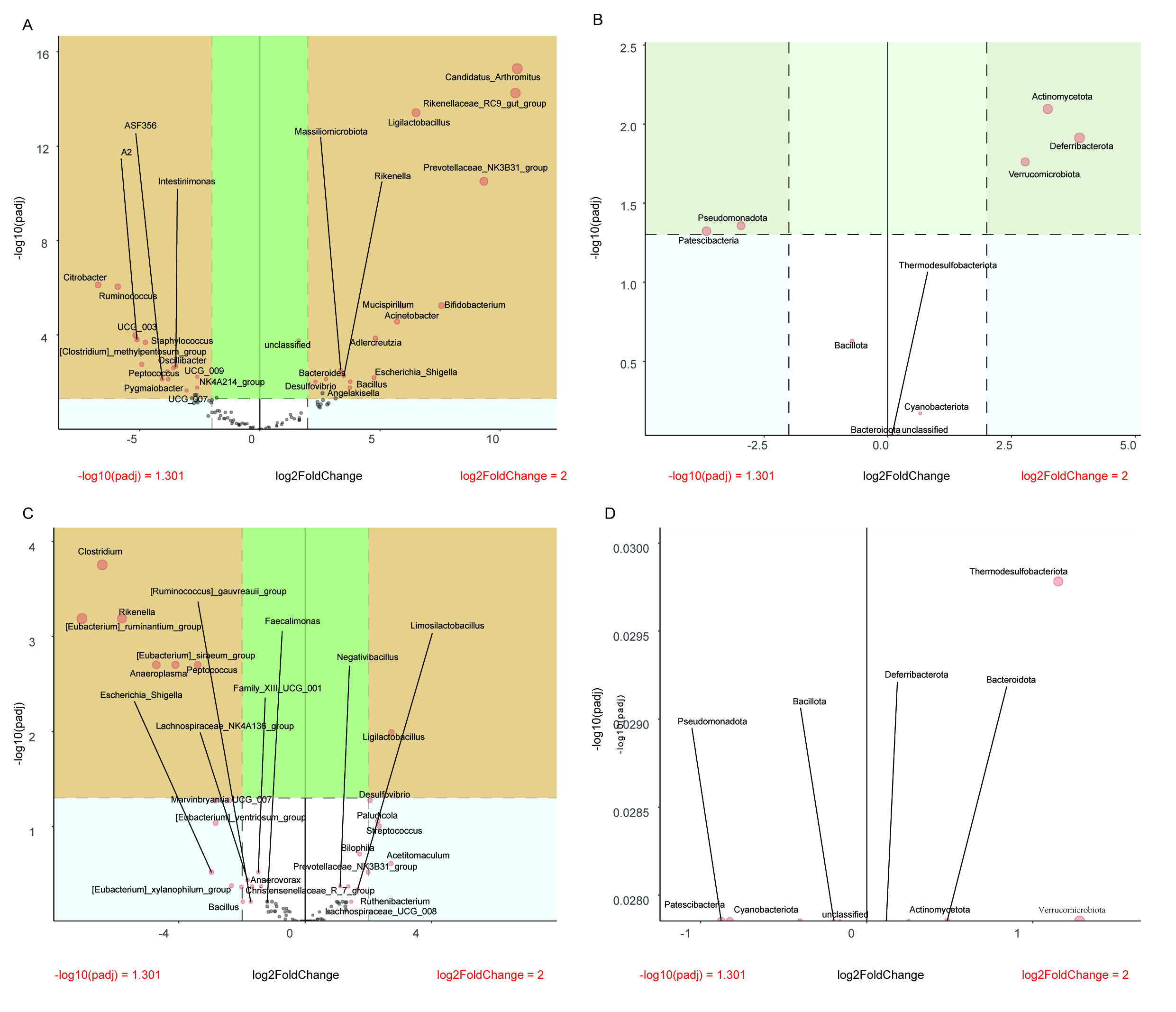

Supplement: Supplementary Figure 4 — Gut microbiota profiling across experimental groups. (A, C) Genus-level and (B, D) phylum-level differential abundance analysis (DESeq2) comparing: (A, B) 3KO-NSCs-treated group vs. VPA-induced ASD model group; (C, D) Normal Control group vs. VPA-induced ASD model group. [file Image4.tif]

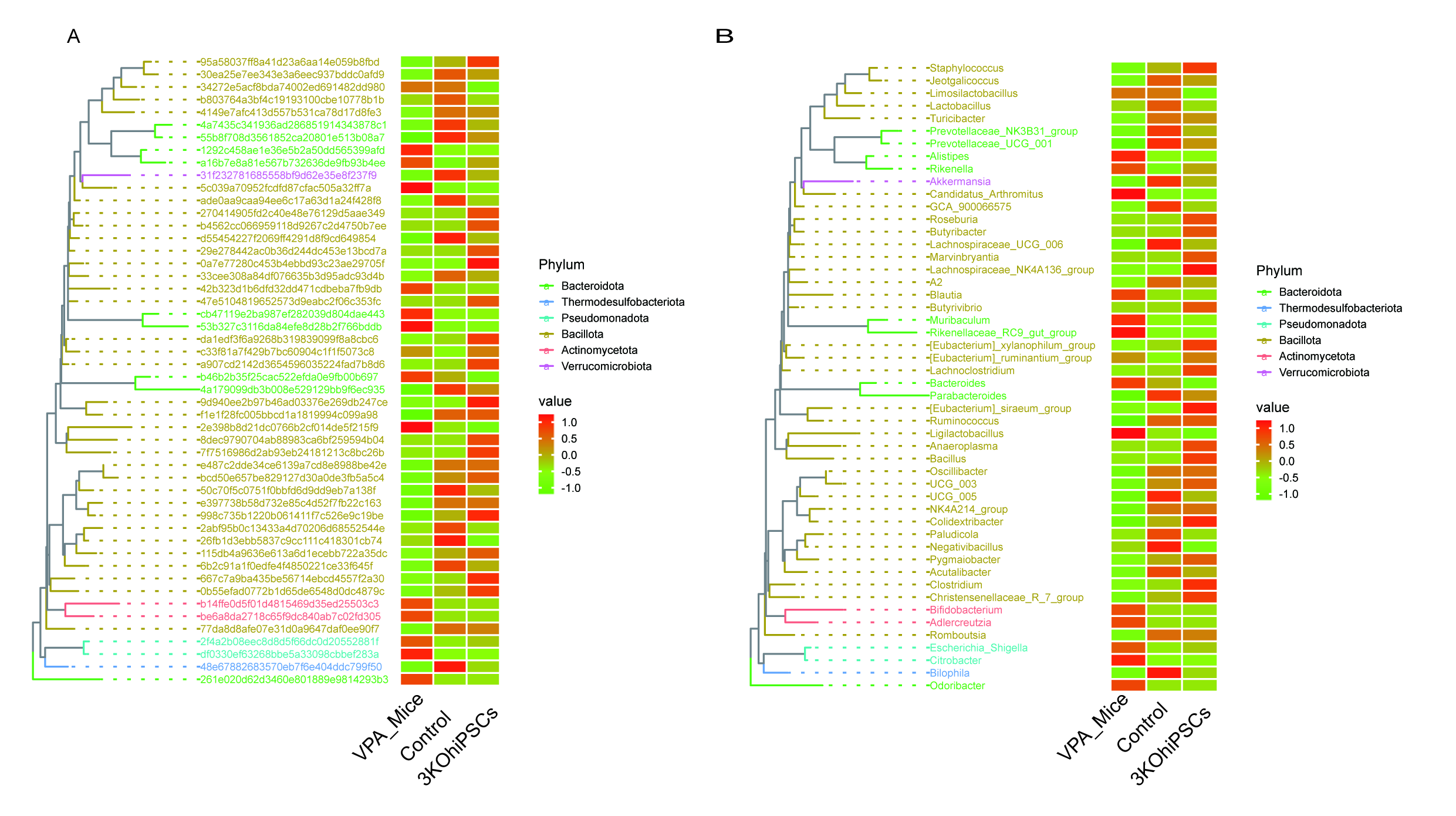

Supplement: Supplementary Figure 5 — Phylogenetic heatmap analysis of gut microbiota composition. (A) Heatmap annotated with taxonomic IDs. (B) Clustered phylogenetic heatmap. [file Image5.tif]

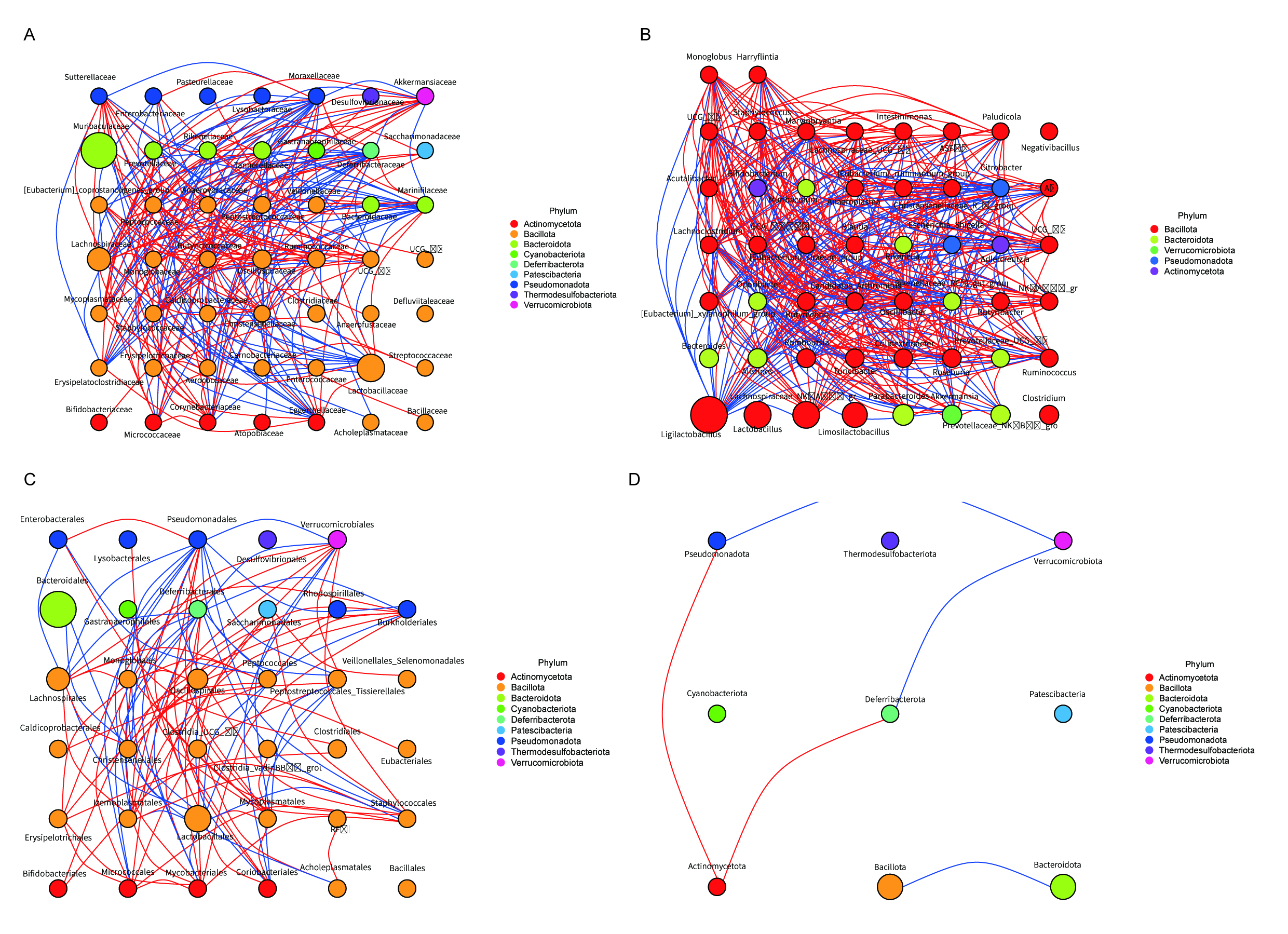

Supplement: Supplementary Figure 6 — Network correlation analysis. (A) Family-level network correlation analysis. (B) Genus-level network correlation analysis. (C) Order-level network correlation analysis. (D) Phylum-level network correlation analysis. [file Image6.tif]
